# Multiomic analysis of microRNA-mediated regulation reveals a proliferative axis involving miR-10b in fibrolamellar carcinoma

**DOI:** 10.1172/jci.insight.154743

**Published:** 2022-06-08

**Authors:** Adam B. Francisco, Matt Kanke, Andrew P. Massa, Timothy A. Dinh, Ramja Sritharan, Khashayar Vakili, Nabeel Bardeesy, Praveen Sethupathy

**Affiliations:** 1 Department of Biomedical Sciences, College of Veterinary Medicine, Cornell University, Ithaca, New York, USA.; 2 Department of Surgery, Boston Children’s Hospital, Boston, Massachusetts, USA.; 3 Department of Medicine, Harvard Medical School, Boston, Massachusetts, USA.

**Keywords:** Oncology, Liver cancer, Molecular genetics, Oncogenes

## Abstract

Fibrolamellar carcinoma (FLC) is an aggressive liver cancer primarily afflicting adolescents and young adults. Most patients with FLC harbor a heterozygous deletion on chromosome 19 that leads to the oncogenic gene fusion, *DNAJB1-PRKACA*. There are currently no effective therapeutics for FLC. To address that, it is critical to gain deeper mechanistic insight into FLC pathogenesis. We assembled a large sample set of FLC and nonmalignant liver tissue (*n* = 52) and performed integrative multiomic analysis. Specifically, we carried out small RNA sequencing to define altered microRNA expression patterns in tumor samples and then coupled this analysis with RNA sequencing and chromatin run-on sequencing data to identify candidate master microRNA regulators of gene expression in FLC. We also evaluated the relationship between *DNAJB1-PRKACA* and microRNAs of interest in several human and mouse cell models. Finally, we performed loss-of-function experiments for a specific microRNA in cells established from a patient-derived xenograft (PDX) model. We identified miR-10b-5p as the top candidate pro-proliferative microRNA in FLC. In multiple human cell models, overexpression of *DNAJB1-PRKACA* led to significant upregulation of miR-10b-5p. Inhibition of miR-10b in PDX-derived cells increased the expression of several potentially novel target genes, concomitant with a significant reduction in metabolic activity, proliferation, and anchorage-independent growth. This study highlights a potentially novel proliferative axis in FLC and provides a rich resource for further investigation of FLC etiology.

## Graphical abstract

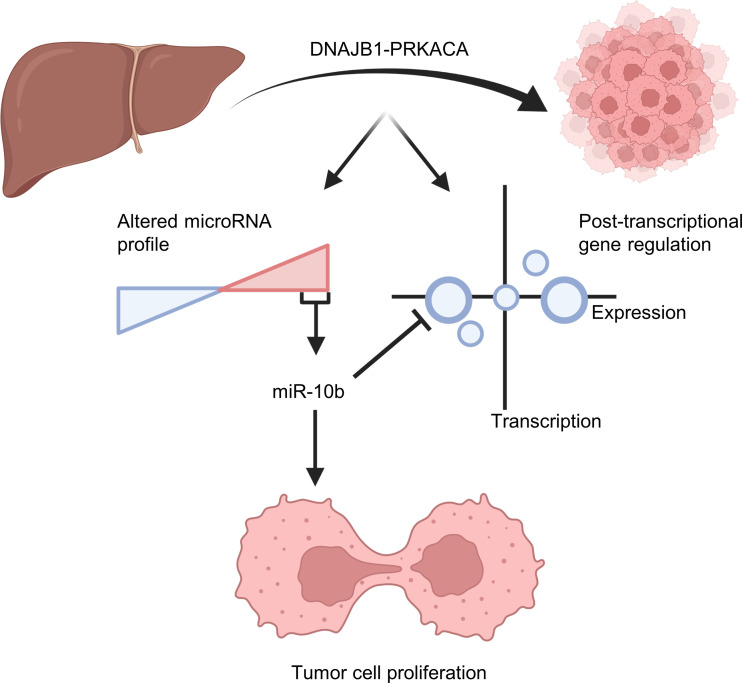

## Introduction

Fibrolamellar carcinoma (FLC) is a rare and aggressive type of liver cancer (1, 2). FLC predominantly afflicts adolescent and young adults who lack predisposing features of other liver cancers, such as alcoholism, obesity, hepatitis or parasitic infections, and chronic exposure to toxins (3). Standard of care for typical liver cancers, referred to as hepatocellular carcinoma, has thus far not proven effective (4, 5). The only current successful treatment option is surgical resection. Unfortunately, detection of FLC often does not occur until later stages of disease progression, and for patients with advanced metastatic cancer for whom resection is not a viable strategy, the prognosis is dismal (6–9). There is a dire need for effective therapies. To work toward this, it is critical to first understand the mechanisms underlying FLC progression.

Nearly all patients with FLC carry a heterozygotic deletion of ~400 kb on chromosome 19 between the genes *DNAJB1* (encoding a small heat-shock factor) and *PRKACA* (encoding a catalytic subunit of the cAMP-dependent protein kinase holoenzyme PKA), which results in the creation of the in-frame fusion gene *DNAJB1-PRKACA* and the eventual expression of the protein chimera DNAJ-PKAc (both abbreviated as “DP”) (10–12). DP is oncogenic in murine livers (13, 14) and thought to be the driver of FLC formation. There is considerable interest in developing chemical inhibitors of DP activity; however, off-target suppression of PKA remains an unfavorable outcome due to the importance of PKA in the normal physiology of critical organs, including the heart (15–17). Genome-scale analysis of FLC tumors has revealed reproducible changes in the expression of many genes (18–20), several of which have been shown to be strongly sensitive to DP activity (21, 22). This finding prompts the hypothesis that genes downstream of DP may represent alternative therapeutic targets.

One class of genes shown to be altered in FLC is microRNAs (22, 23), which are ~21 nt small RNAs that negatively regulate gene expression at the posttranscriptional level (24). Two initial studies identified dysregulated microRNAs but were underpowered in terms of the number of FLC tumor samples (18 in 1 study and 7 in another study; refs. 22, 23). In this study, we performed small RNA-Seq analysis in the largest set of FLC and nonmalignant samples to date (*n* = 52) and identified a set of significantly upregulated microRNAs in both primary and metastatic tumors. We found that miR-10b is among the most dramatically upregulated in FLC. There was little difference overall between primary and metastatic microRNA expression profiles. We then leveraged our published FLC chromatin run-on sequencing (ChRO-Seq) data to identify the transcriptional start sites and quantify transcriptional activity of the significantly upregulated microRNAs; this process revealed that miR-10b is the most transcriptionally activated microRNA locus in FLC. We then performed an integrative analysis of ChRO-Seq and RNA-Seq data sets to identify those genes subject primarily to posttranscriptional regulation (PTR) in FLC. Among the PTR genes that are downregulated in FLC, we observed a significant enrichment of predicted binding sites for miR-10b.

We found that the expression and activity of DP is sufficient to induce the upregulation of miR-10b in multiple human, but not mouse, cell models of FLC, which points to important species-specific differences in terms of the downstream effects of DP. We then performed functional analysis of miR-10b in 2 FLC cell lines, independently derived from a patient-derived xenograft (PDX). After inhibition of miR-10b, we observed a modest but significant reduction in cell metabolism, anchorage-independent growth, and proliferation, which associates with a significant increase in the expression of potentially novel candidate target genes of miR-10b: *FANCC*, *SUN2*, and *TRIM35*. All 3 of these genes have been implicated as tumor suppressors in other cancer types (25–32). Overall, our results reveal a regulatory axis in FLC involving miR-10b to promote cellular proliferation.

## Results

### MicroRNA profiling in what is potentially the largest FLC and nonmalignant liver sample set to date.

**Figure 1:**
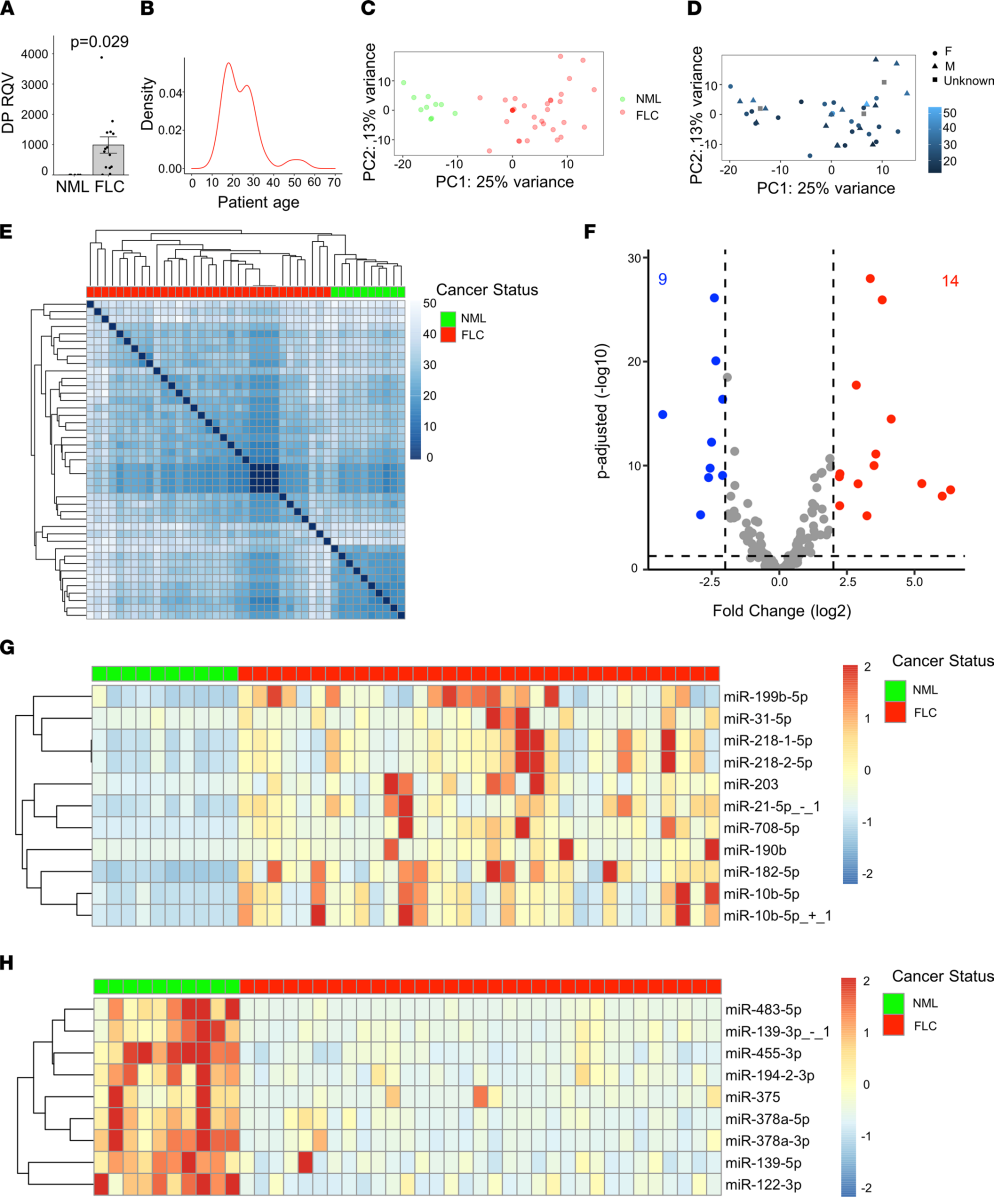
miR-10b is among the most upregulated microRNAs in FLC. (**A**) qPCR showing the relative quantitative value (RQV) of DP in a subset of FLC samples (*n* = 15) compared with NML samples (*n* = 6). Data points represent individual patient samples. Cycle threshold values can be found in Supplemental Table 1. (**B**) Patient age distribution across FLC samples used in this study for which age is known (*n* = 35). (**C**) Principal component analysis of VST normalized counts for the NML (*n* = 10) and FLC (*n* = 33) data sets. The percent of variation explained is indicated for component 1 (*x* axis) and component 2 (*y* axis). NML and FLC samples are colored green and red. (**D**) Principal component analysis plot in which the patient age and sexual phenotype information are overlaid. Female, male, and unreported patients are indicated by circles, triangles, and squares, respectively. The color intensity, from dark to light, indicates increasing patient age at the time of surgery. (**E**) Unsupervised hierarchical clustering of the Euclidean distances among samples was calculated based on VST normalized counts. FLC and NML samples are indicted by red and green boxes. (**F**) Volcano plot showing microRNAs that are significantly differentially expressed (average normalized counts > 1000 in either NML or FLC, coefficient of variance < 2 across FLC samples). Dashed lines represent the log_2_ FC of expression –2/+2 (vertical) and adjusted *P* = 0.05 (horizontal). Up- or downregulated microRNAs are colored red or blue, respectively. (**G** and **H**) Heatmaps showing the normalized expression of up- or downregulated microRNAs (in rows) in each patient sample (in columns). Expression is scaled by row with a max/min of +2/–2 shown. *P* values are calculated by 2-tailed Student’s *t* test.

Tissue samples from patients with FLC were acquired though a collaboration with the Fibrolamellar Cancer Foundation (FCF) biobank, resulting in a substantially expanded set (*n* = 52, *n* = 47 after removal of samples acquired from the same patient at a different time) relative to our prior analysis (22), which included only 18 samples. Patient samples were obtained at the time of surgical procedures, frozen, and stored at –80°C. When possible, nonmalignant liver (NML) adjacent to the FLC tumors was also obtained. We confirmed the presence of the DP oncogene in each sample by quantitative PCR (qPCR), detection of the fusion transcript in RNA-Seq data, or Western blot (Supplemental Table 1; supplemental material available online with this article; https://doi.org/10.1172/jci.insight.154743DS1). The expression of DP in samples analyzed by qPCR (Figure 1A) was, on average, ~1000-fold higher in FLC than in NML tissue. We also determined that the median age of the patients in our data set was 21 years old (Figure 1B), consistent with the notion that FLC is primarily an adolescent and young adult cancer.

**Figure 2:**
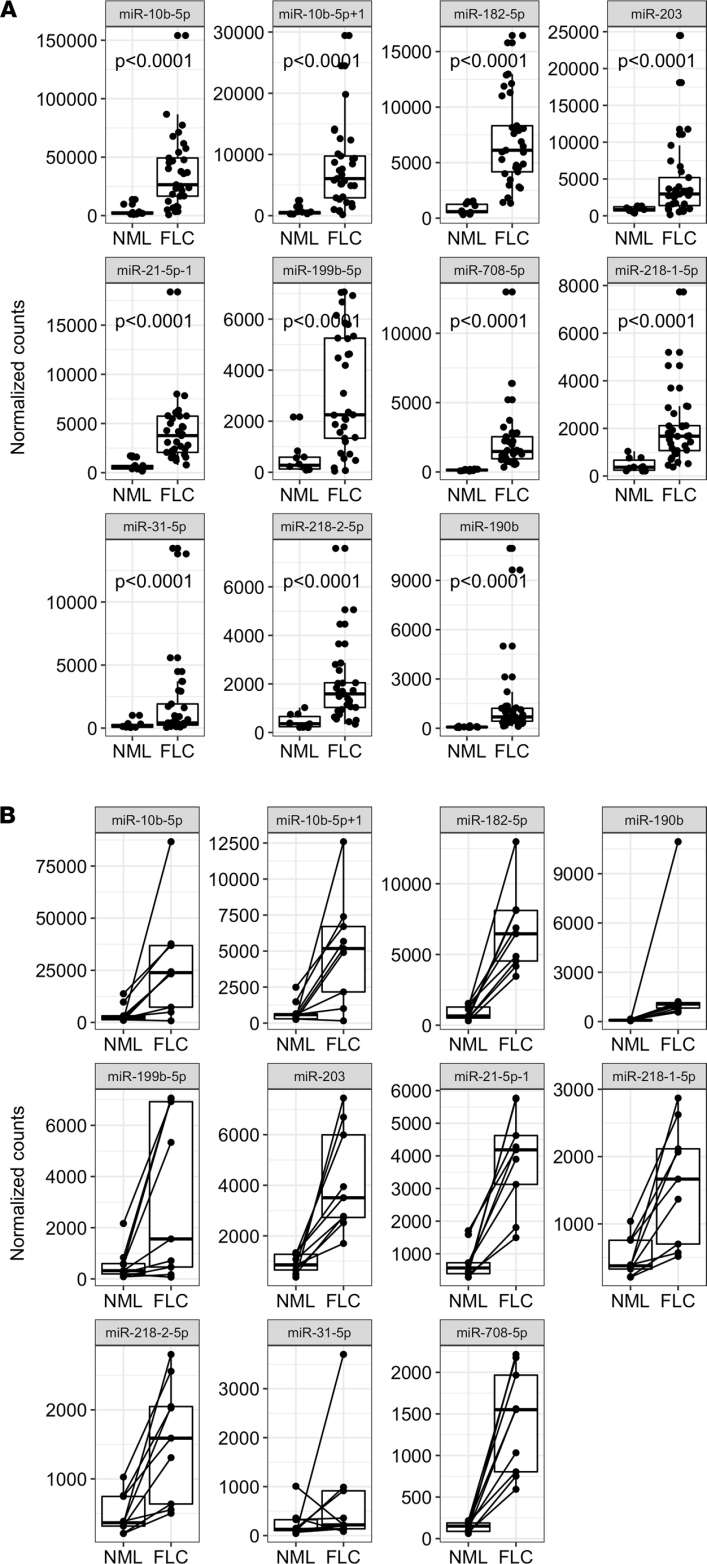
Expression levels of the significantly upregulated microRNAs in all FLC patient samples. (**A**) The normalized expression of each upregulated microRNA is shown as individual box plots for NML and FLC. Samples are plotted as individual points. (**B**) Expression of upregulated microRNAs among FLC patients with matched NML samples (*n* = 9). The matched NML/FLC samples are indicated with a line linking the 2 data points. Each data point represents a patient sample. *P* values are calculated by 2-tailed Student’s *t* test.

We extracted RNA from each patient sample and proceeded with small RNA-Seq. After ensuring that all samples had a sufficient number of mapped reads (>1 million reads; Supplemental Table 2), we quantified microRNA expression using miRquant 2.0 (33). Principal component analysis (PCA) revealed that the microRNA expression profiles were sufficient to stratify samples according to NML and FLC status (Figure 1C), but not age or sex (Figure 1D), indicating that microRNA differences are due to tumor biology. Unsupervised hierarchical clustering analysis confirmed that FLC samples stratified completely separately from NML samples (Figure 1E). We then directly compared microRNA profiles between FLC and NML to identify significantly differentially expressed microRNAs (*n* = 23; Figure 1F). For each of these microRNAs, we calculated the coefficient of variance (CoV) to measure the level of expression variability across FLC samples. After setting a maximum CoV threshold of 2.0, we identified 20 microRNAs of interest in FLC (11 upregulated, 9 downregulated). The expression level of each up- or downregulated microRNA is shown for each patient (Figure 1, G and H). The downregulated set includes miR-375, which we reported previously as a likely tumor suppressor in FLC (22). Due to our previous work on tumor suppressor microRNAs in FLC (22), we focused the remainder of this study on the upregulated microRNAs, which represent candidate oncogenes. The upregulated set comprises miR-199b-5p, miR-31-5p, miR-218-1-5p, miR-218-2-5p, miR-203, miR-21-5p+1, miR-708-5p, miR-190b, miR-182-5p, miR-10b-5p, and miR-10b-5p+1, where +1 denotes a 1-nucleotide shift in the 5’-end start position relative to the canonical microRNA sequence (Figure 2A). Among these, miR-10b was the most abundant in FLC tumors (>5-fold more abundant on average than the next-most abundant microRNA).

Within our patient sample set, we have 9 matched samples to compare the microRNA expression in FLC tumor with adjacent NML tissue. The 11 microRNAs upregulated in the full data set exhibited very similar expression trends in this matched data set (Figure 2B).

### Expression of microRNAs in primary versus metastatic FLC.

**Figure 3:**
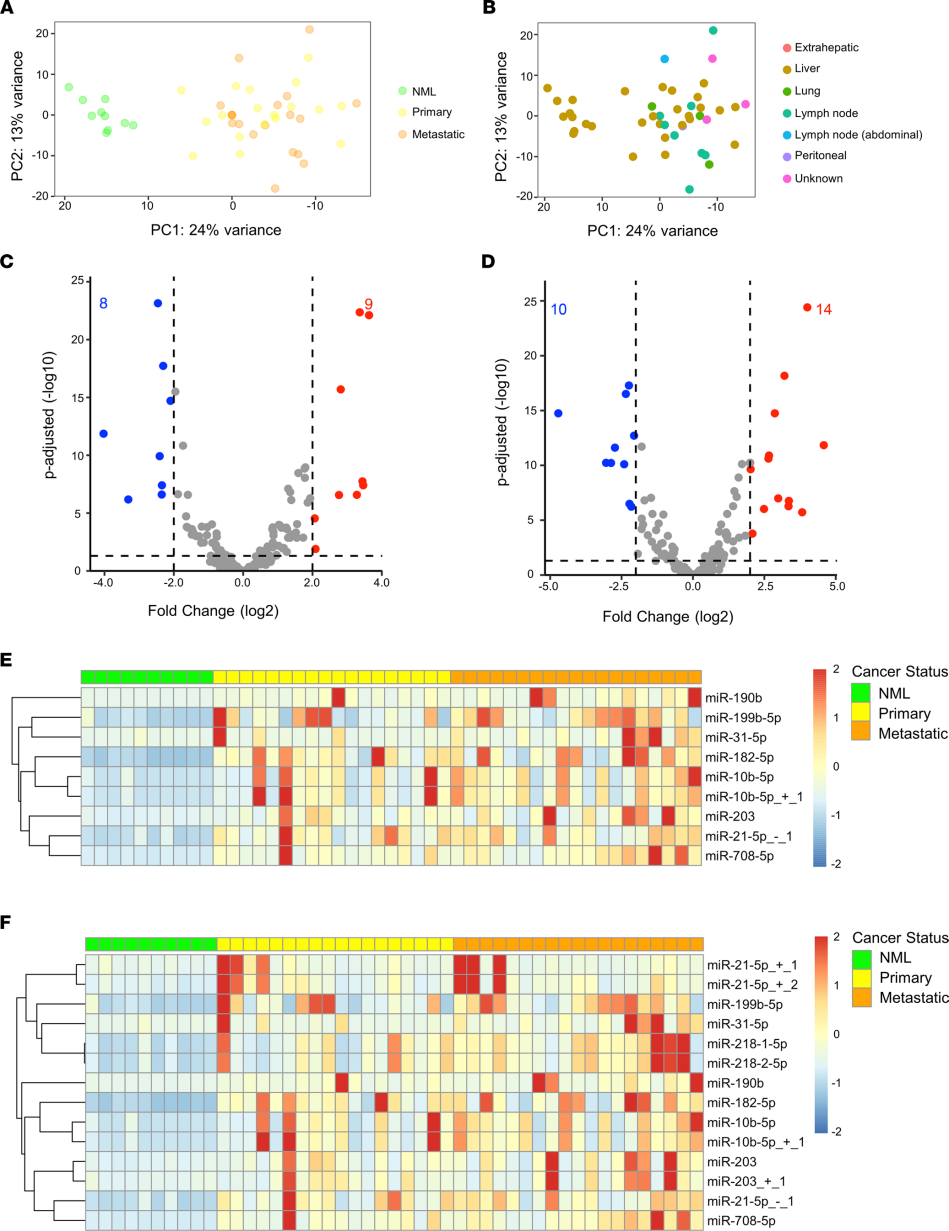
miR-10b is among the most upregulated microRNAs in both primary and metastatic FLC. (**A**) Principal component analysis of VST normalized counts with tumor type information overlaid. NML (*n* = 10), primary (*n* = 18), and metastatic (*n* = 19) samples are shown in green, yellow, and orange, respectively. (**B**) Principal component analysis of VST normalized counts with metastatic location information overlaid. Extrahepatic, liver, lung, lymph node, peritoneal, and unknown locations are shown red, brown, green, teal, blue, and purple, respectively. (**C**) Volcano plot showing microRNAs that are significantly differentially expressed in primary FLC versus NML (average normalized counts > 1000 in either NML or primary FLC). Dashed lines represent the log_2_ FC of expression –2/+2 (vertical) and adjusted *P* = 0.05 (horizontal). Up- or downregulated microRNAs are colored red or blue, respectively. (**D**) Volcano plot showing microRNAs that are significantly differentially expressed in metastatic FLC versus NML; analysis criteria identical to **C**. Dashed lines represent the log_2_ FC of expression –2/+2 (vertical) and adjusted *P* = 0.05 (horizontal). Up- or downregulated microRNAs are colored red or blue, respectively. (**E**). Heatmap showing the expression of microRNAs upregulated in primary FLC versus NML. MicroRNAs are listed in rows and individual patients are listed in columns. Expression is scaled by row with a max/min of +2/–2 shown. (**F**) Heatmap showing the expression of upregulated microRNAs in metastatic FLC versus NML; sample arrangement is identical to **E**. Expression is scaled by row with a max/min of +2/–2 shown.

Next, we analyzed the differential expression of microRNAs in primary or metastatic FLC tumor tissue relative to NML. Primary tumor samples were all hepatic in origin, and metastatic samples were from liver, lung, lymph nodes, pancreas, or peritoneum (Supplemental Table 1). The expression of microRNAs did not stratify primary from metastatic samples according to PCA (Figure 3A). We also observed no specific clustering based on different sites of FLC malignancy (Figure 3B), suggesting that FLC maintains highly consistent differential expression of microRNAs irrespective of tumor type or metastatic location. When comparing only primary tumors (*n* = 18) to NML (*n* = 10), we identified 9 upregulated and 8 downregulated microRNAs (Figure 3, C and E). We performed an identical analysis comparing only metastatic tumor tissue (*n* = 19) to NML (*n* = 10) and found that 14 microRNAs were upregulated and 10 were downregulated (Figure 3, D and F). These microRNAs comprise all of those identified in the primary versus NML analysis (Figure 3C) and all-FLC versus NML analysis (Figure 1G), as well as 3 additional microRNAs, which are isoforms of canonical miR-21 and miR-203, referred to as isomiRs. Upon further analysis of the 14 upregulated microRNAs, none were found to be significantly differentially expressed in primary versus metastatic FLC tissue (Supplemental Figure 1).

### Expression of microRNAs in FLC compared with other cancer types.

**Figure 4:**
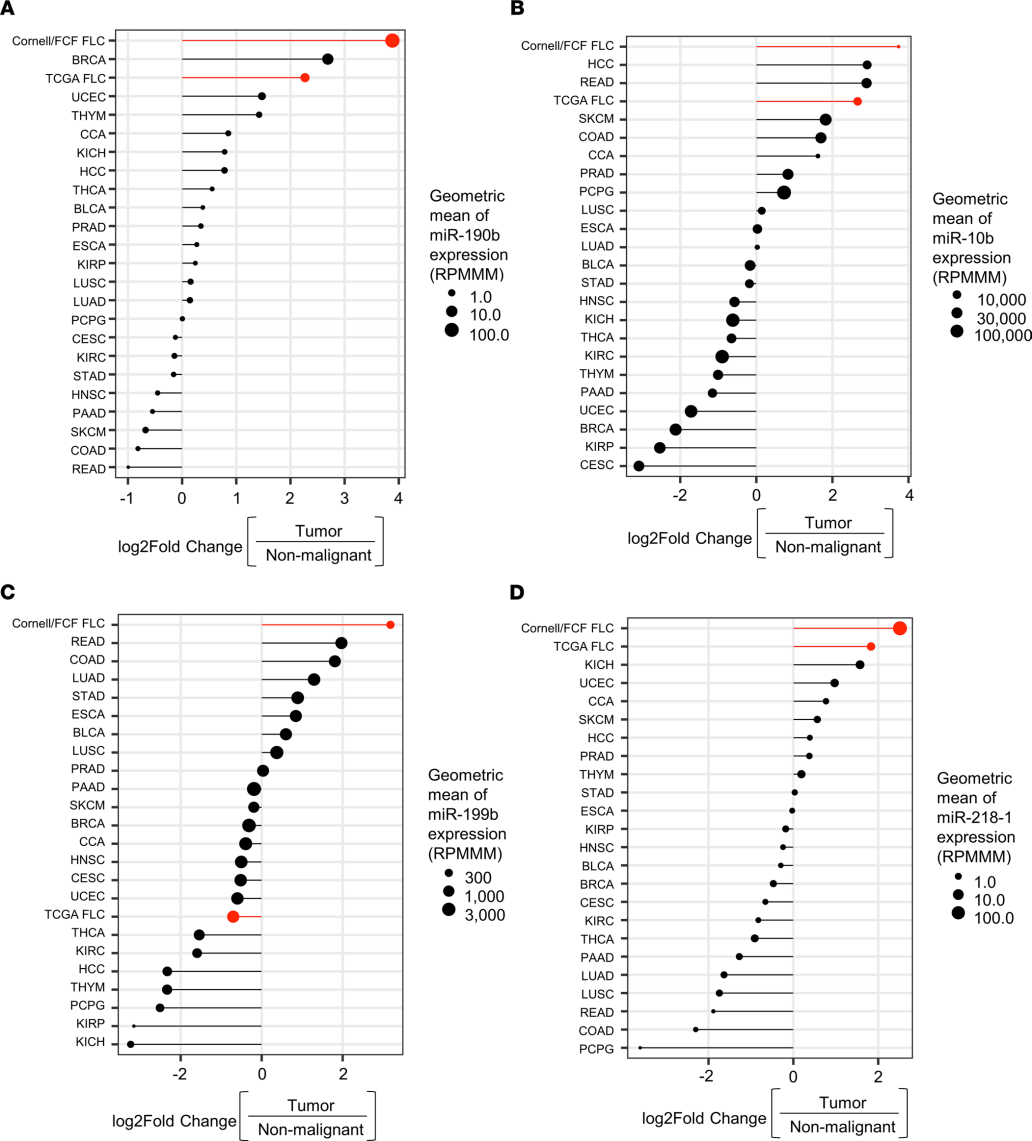
FLC microRNA expression compared with other cancer types. (**A**–**D**) Log_2_ FC expression of the 4 most upregulated microRNAs in FLC (after removing isomiRs) within TCGA. The size of each circle represents the geometric mean of microRNA expression in each tumor type. Each tumor type is ranked on the *y* axis by the log_2_ FC of the geometric mean of tumor expression relative to nontumor expression. The FLC sample set used in this study (Cornell/FCF) and the FLC sample set available from TCGA (*n* = 6) are highlighted in red. BLCA, bladder urothelial carcinoma; BRCA, breast invasive carcinoma; CESC, cervical squamous cell and endocervical adenocarcinoma; CCA, cholangiocarcinoma; COAD, colon adenocarcinoma; Cornell/FCF FCL, fibrolamellar carcinoma samples analyzed in this study; ESCA, esophageal carcinoma; HCC, hepatocellular carcinoma; HNSC, head and neck squamous cell carcinoma; KICH, kidney chromophobe; KIRC, kidney renal papillary cell carcinoma; KIRP, kidney renal clear cell carcinoma; LUAD, lung adenocarcinoma; LUSC, lung squamous cell carcinoma, PAAD, pancreatic adenocarcinoma; PCPG, pheochromocytoma and paraganglioma; PRAD, prostate adenocarcinoma; READ, rectum adenocarcinoma; SKCM, skin cutaneous melanoma; STAD, stomach adenocarcinoma; TCGA FLC; fibrolamellar carcinoma; THCA, thyroid carcinoma; THYM, thymoma; UCEC, uterine corpus endometrial carcinoma; RPMMM, reads per million mapped to microRNAs.

Next, we compared the expression of the 11 upregulated microRNAs in FLC (9 when discounting isomiRs) (Figure 1G) across all 23 cancer types available in The Cancer Genome Atlas (TCGA) for which small RNA-Seq has been performed and nonmalignant tissue controls exist. We previously identified (19) 6 FLC samples within TCGA (designated TCGA FLC), which we report here separately from the set of FLC samples used in this study (designated as Cornell/FCF FLC). We found that only 4 microRNAs (miR-190b, miR-10b, miR-199b, and miR–218-1) exhibited a level of increased expression in the Cornell/FCF FLC tumors that was greater than the increase observed in any other cancer type (Figure 4, A–D). We also noted that there were 3 microRNAs (miR-10b, miR-708, and miR–218-2) that exhibited concordant increases between the Cornell/FCF FLC and TCGA FLC sets; increases in these microRNAs in the Cornell/FCF FLC set were most similar to gastrointestinal cancers (in particular, colon adenocarcinoma [COAD] and rectal adenocarcinoma [READ]) (Supplemental Figure 2, A–E).

### Transcriptional activity at microRNA loci in FLC.

**Figure 5:**
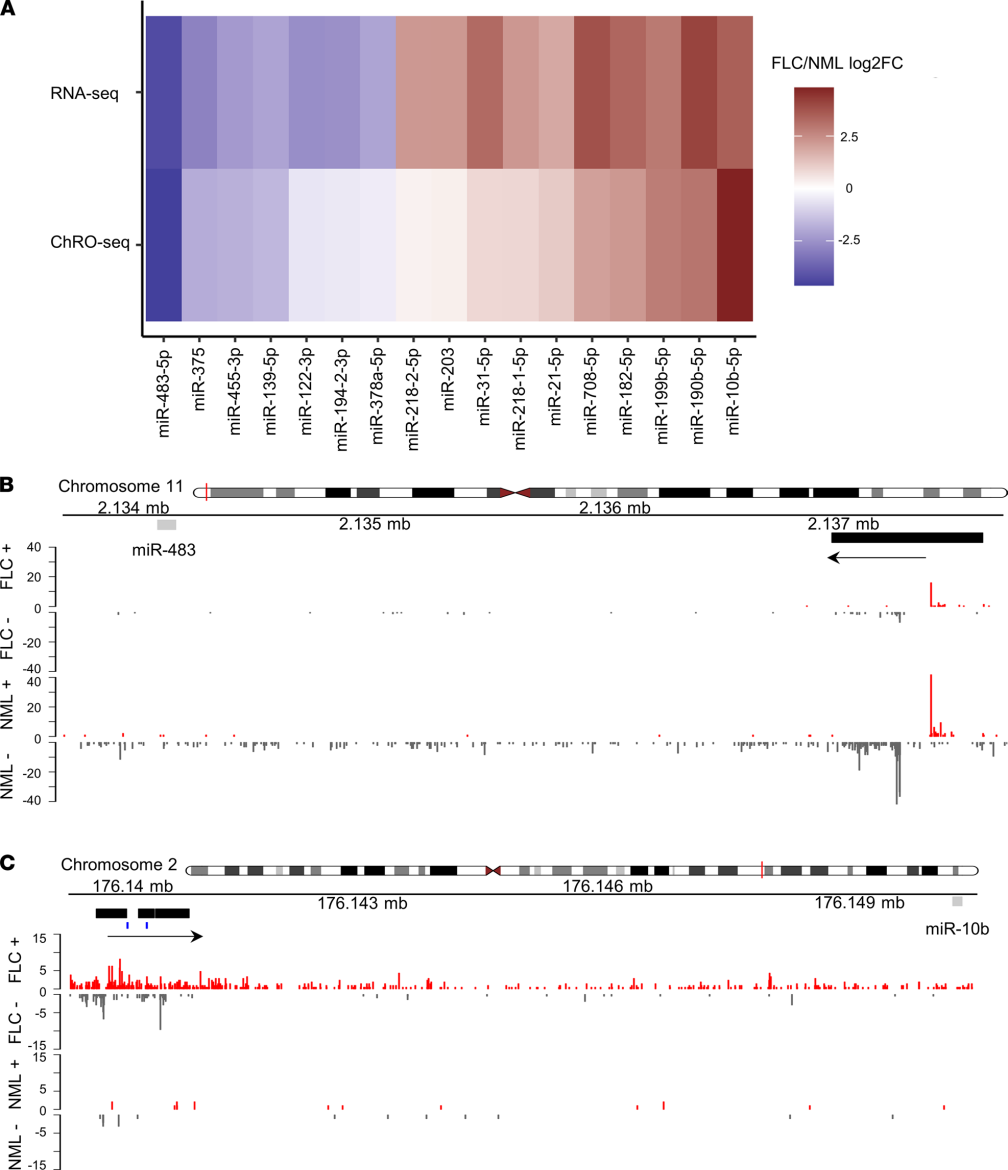
miR-10b is the most transcriptionally activated microRNA in FLC. (**A**) Gene expression (RNA-Seq signal, *n* = 19, top row) or transcriptional activity (ChRO-Seq signal, *n* = 13, bottom row) for the 17 significantly differentially expressed microRNAs (after removing isomiRs) is shown as the log_2_ FC in FLC versus NML. (**B**) miR–483-5p and (**C**) miR–10b-5p genome browser tracks showing normalized ChRO-Seq signal. The upper and lower panel show the activity in FLC and NML. Activity on the plus and minus strand are shown in red and gray, respectively. The mature microRNA sequence is shown as a gray rectangle, the promoter region of the miRNA is shown as a black rectangle, and the direction of transcription is identified by an arrow. Predicted FOXQ1 bindings sites in the miR-10b promoter are identified by blue dashes.

Aberrant microRNA expression in cancer can be due to changes in transcriptional and/or PTR (34). Therefore, we next sought to determine which of the microRNA changes in FLC are driven by transcription. We used our previously published (21) ChRO-Seq data across 13 FLC samples to identify the promotor regions of all microRNAs differentially expressed in FLC (*n* = 20; after removal of isomiRs, *n* = 17) (Figure 1, G and H). We then used the same ChRO-Seq data to quantify the level of transcription from each of the microRNA promoters in FLC versus NML. We observed an overall concordance between the fold-change in transcription (ChRO-Seq) and the fold-change in expression (small RNA-Seq) for most of the 17 microRNAs (Figure 5A). In all cases, transcriptional activation or repression led directly to increased or decreased expression, respectively. However, for a few microRNAs, such as miR-203 and miR–378a-5p, the amplitude of the fold change in expression was much greater than the fold-change in transcription, suggesting that these microRNAs may be altered in FLC primarily by posttranscriptional mechanisms.

MiR–483-5p (Figure 5B) and miR-10b (Figure 5C) exhibited the greatest decrease and increase in transcription, respectively, in FLC relative to NML (Figure 5A). It has been reported in other cancer types (35–37) that miR-10b is transcriptionally activated by the Twist1 transcription factor, and we identified a ~3-fold increase of *TWIST1* expression in FLC (Supplemental Figure 3A). However, we observed that *TWIST1* was negatively correlated to DP expression (Supplemental Figure 3B). We then performed an unbiased search of the miR-10b promoter region using the online resource Find Individual Motif Occurrences (FIMO) (38), which revealed high-confidence binding sites for FOXQ1, as defined by the JASPER transcription factor binding profile database (39). We identified a ~5-fold increase in *FOXQ1* expression in FLC and a significant positive correlation to DP expression (Supplemental Figure 3, C and D) across 19 FLC samples for which we have matched RNA-Seq and small RNA-Seq data.

### DP promotes miR-10b expression in human but not mouse cell models.

**Figure 6:**
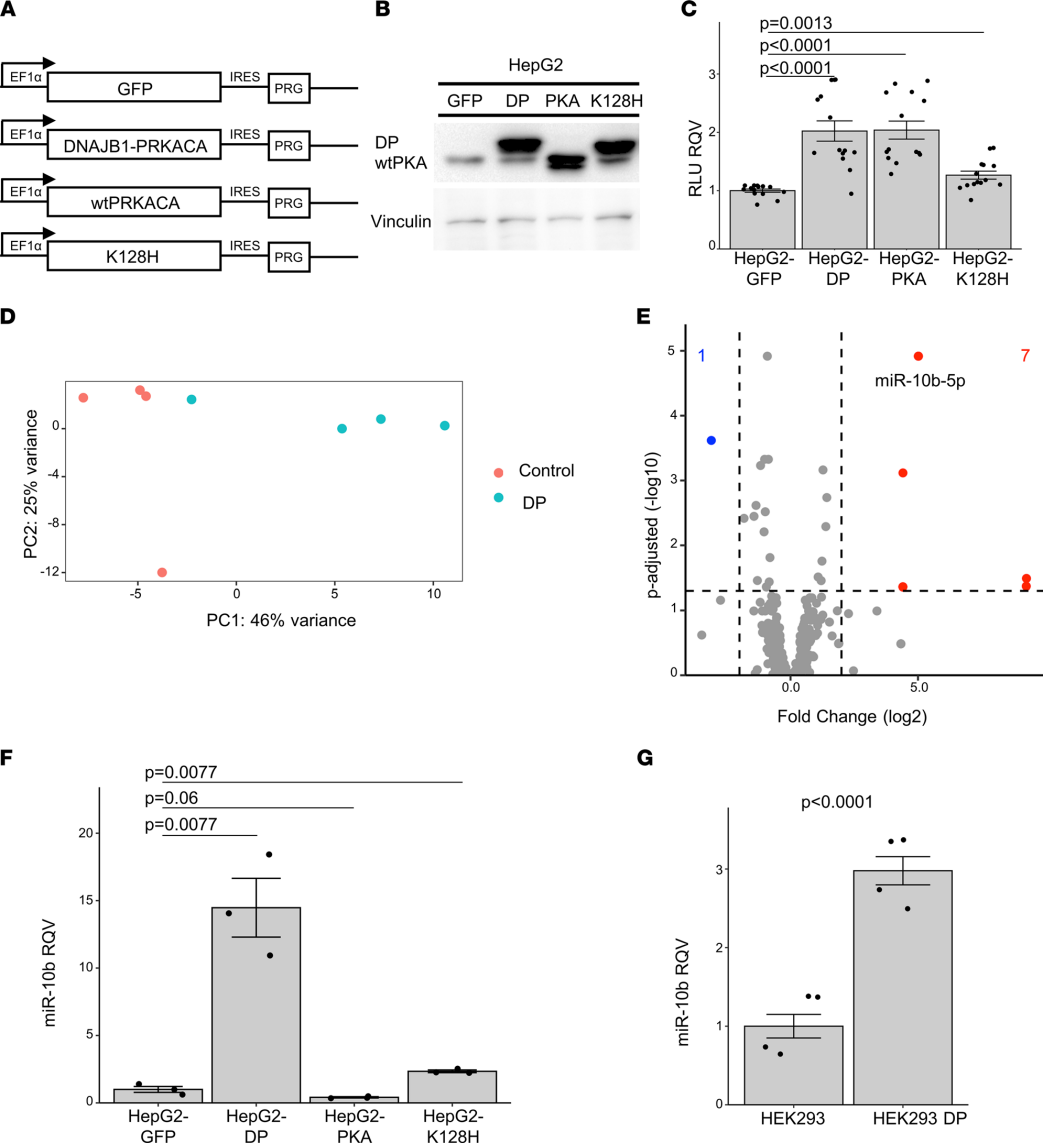
DP activity promotes the expression of miR-10b in human but not mouse models. (**A**) Diagram of expression cassettes for lentiviral constructs. EF1α; human EF1α gene promoter; GFP, green fluorescent protein; DNAJB1-PRCACA, FLC fusion oncogene; WT PRKACA, protein kinase A catalytic α subunit; DNAJB1-PRCACA K128H, FLC fusion oncogene containing a lysine-to-histidine substitution at amino acid position 128; IRES, internal ribosome entry site; PRG, puromycin resistance gene. (**B**) Protein expression in HepG2 cell lines detected with a protein kinase A catalytic α subunit (PKA) antibody. WT PKAc and DP are identified. Lane 1, HepG2-GFP; lane 2, HepG2-DP; lane 3, HepG2-PKA; lane 4, HepG2-K128H. Vinculin expression for loading control is shown in the lower panel. Uncropped immunoblot is shown in Supplemental Figure 4, A and B. (**C**) Luciferase relative light units (RLU), proportional to viable cells, is shown relative to the HepG2-GFP cell line (2 trials, *n* = 14 each condition). (**D**) Principal component analysis of the log-transformed normalized counts from small RNA-Seq of HepG2-DP (DP, *n* = 3) and HepG2-GFP (control, *n* = 3) samples, in blue and red, respectively. (**E**) Volcano plot showing the differentially expressed microRNAs in HepG2-DP relative to HepG2-GFP (only microRNAs with average HepG2-DP or HepG2-GFP expression > 100 shown). Dashed lines represent the log_2_ (fold change) of expression –2/+2 (vertical) and adjusted *P* = 0.05 (horizontal). Up- or downregulated microRNAs are colored red or blue, respectively. (**F**) qPCR showing the relative quantitative value (RQV) for miR-10b expression in HepG2-GFP, HepG2-DP, HepG2-PKA, and HepG2-K128H cell lines (*n* = 3 each). (**G**) qPCR for showing the RQV for miR-10b expression in HEK293 and HEK293-DP cell lines (*n* = 4 each). In all assays, each dot represents the average signal of a biological replicate. *P* values are calculated by 2-tailed Student’s *t* test. *P* values reported in **C** and **F** were adjusted for multiple testing correction post hoc by the Benjamini-Hochberg method.

Having identified miR-10b as: (a) one of the most differentially expressed microRNAs in FLC (Figure 1G), (b) the most highly expressed among the upregulated microRNAs in FLC (Figure 2A), (c) aberrantly expressed in both primary and metastatic FLC tissue (Supplemental Figure 1A), and (d) the most transcriptionally activated microRNA in FLC compared with NML (Figure 5A), we next asked if miR-10b is directly responsive to the expression and activity of DP. We constructed lentiviral vectors (21, 22) (Figure 6A) for transduction and ectopic expression of enhanced green fluorescent protein (GFP), DP, protein kinase A catalytic α subunit (PKA), or a catalytically inactive form of DP carrying a lysine-to-histidine amino acid substitution at position 128 (K128H). The protein expression of WT PKA is shown in the GFP samples (Figure 6B and Supplemental Figure 4, A and B), as well as in the 3 DP-expressing clones (Figure 6B and Supplemental Figure 4, A and B), migrating at approximately 40 kD. As expected, there was increased expression of WT PKA in the HepG2 line overexpressing PKA (Figure 6B and Supplemental Figure 4, A and B), and DP protein (identified by the higher molecular band migrating at approximately 46 kD) was detectable only in the HepG2 lines overexpressing DP or DP-K128H (Figure 6B and Supplemental Figure 4, A and B). We observed that the overexpression of DP or WT PKA led to a significant increase in the number of metabolically active, viable cells relative to HepG2-GFP cells (Figure 6C and Supplemental Figure 4C), whereas the overexpression of K128H had a much milder impact on the number of viable cells (Figure 6C and Supplemental Figure 4C).

Having established that the expression of DP affects cell abundance, we next performed small RNA-Seq in the HepG2-GFP and HepG2-DP cell lines to identify changes in microRNA expression mediated by DP. PCA analysis showed that microRNA profiles stratified replicates of HepG2-GFP from HepG2-DP samples (Figure 6D). Differential expression analysis identified 8 microRNAs that were significantly altered in HepG2-DP relative to HepG2-GFP cells (Figure 6E). Among these, miR-10b was the most significantly upregulated (~30-fold). Other microRNAs that we had identified as upregulated in FLC tumors (Figure 1G) were not altered by DP in this cell model (Figure 6E). We confirmed by qPCR the dramatic upregulation of miR-10b in HepG2-DP cells, and we showed that miR-10b was not changed in HepG2-PKA and only modestly changed in HepG2-K128H cells (Figure 6F). These data demonstrate that miR-10b increase was specific to DP overexpression. We also analyzed by qPCR the expression of 2 other microRNAs that were significantly upregulated in FLC, miR-182 and miR-21 (Figure 1G), and found that there was no significant induction in the HepG2-DP cell line (Supplemental Figure 4D). These data suggest that DP activity had a very robust effect on miR-10b and that the upregulation of other microRNAs in FLC may be contextual to the unknown cell of origin.

We then turned to another human cell model, HEK293-DP, which are HEK293 cells in which the ~400 kb deletion found in human FLC patients was recreated by CRISPR/Cas9 (40), leading to the expression of DP. We observed by qPCR that miR-10b was significantly elevated (~3-fold) in HEK293-DP cells relative to WT HEK293 cells (Figure 6G). We next analyzed a single tissue sample from a patient with intraductal oncocytic papillary neoplasm (IOPN) of the pancreas, which is the only other cancer type in which DP has been detected (41), and found that miR-10b was elevated (~1.5 fold) compared with the nonmalignant pancreas tissue (Supplemental Figure 4E). We also assessed 3 different murine-based models of FLC: (a) AML-12-DP cells (42), (b) TIB-75-DP cells, and (c) DP-expressing mouse (14); DP was expressed by in vivo transposition. Surprisingly, we found that, in all 3 cases, the expression of miR-10b was not increased and, in some cases, was actually reduced by the presence of DP (Supplemental Figure 4, F–H). These findings reveal that DP regulates miR-10b oppositely in human versus mouse cells; therefore, mouse models of FLC are likely not appropriate for studying the role of miR-10b in FLC.

### Identification of genes subject to PTR by microRNAs in FLC.

**Figure 7:**
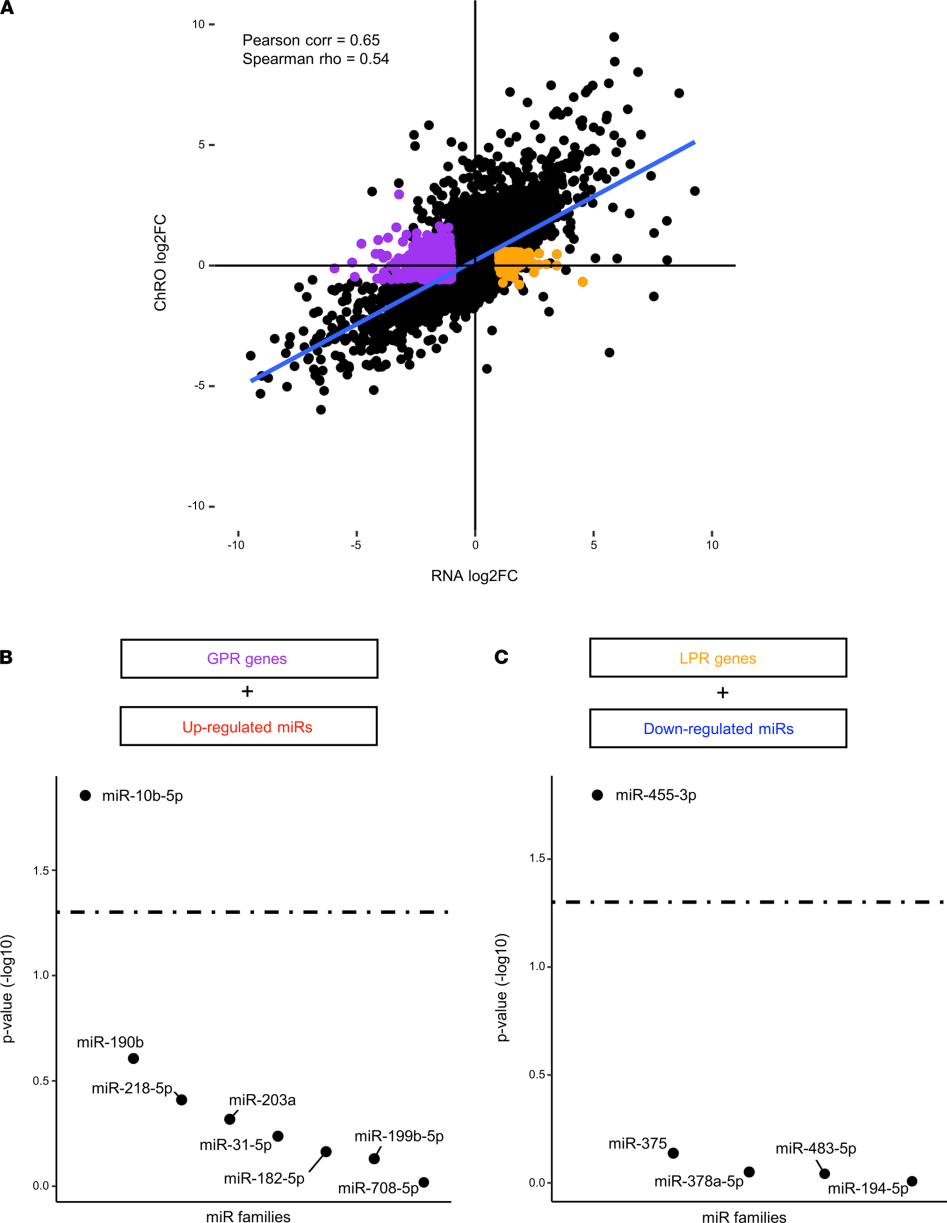
miR-10b and miR-455 are master regulators of gene expression in FLC. (**A**) Scatterplot showing the log_2_ FC of RNA-Seq normalized reads on the *x* axis and the log_2_ FC of ChRO-Seq normalized reads on the *y* axis for genes in FLC relative to NML. Those genes subject primarily to gain of PTR (normalized reads > 1000, RNA-Seq log_2_ FC < 1, RNA-Seq *P*_adj_ < 0.05, ChRO-Seq log_2_FC < ±0.59, ChRO-Seq *P*_adj_ > 0.2) are highlighted in purple, and genes subject primarily to loss of PTR (normalized reads > 1000, RNA-Seq log_2_ FC > 1, RNA-Seq *P*_adj_ < 0.05, ChRO-Seq log_2_FC < ±0.59, ChRO-Seq *P*_adj_ > 0.2) are highlighted in orange. (**B**) Ranked –log_10_ (*P* value) of miRhub simulation results. Gain of PTR genes were examined for enrichment of binding sites for microRNAs upregulated in FLC (only those microRNAs with predictions in TargetScan included). The dashed line represents *P* = 0.05. (**C**) Ranked –log_10_ (*P* value) of miRhub simulation results. Loss of PTR genes was examined for enrichment of binding sites for microRNAs downregulated in FLC (only those microRNAs with predictions in TargetScan included). The dashed line represents *P* = 0.05.

To determine which genes expressed in FLC are likely regulated by microRNAs, we performed an integrative analysis of RNA-Seq and ChRO-Seq data. We first showed across all genes that the change in transcription was highly correlated with the change in mRNA expression in FLC versus NML (Figure 7A), indicating that most gene expression changes in FLC were strongly driven by transcription. Nonetheless, some genes did exhibit robust changes in expression in FLC versus NML despite little to no change in transcription, and these are top candidates for PTR by microRNAs. We defined 788 genes that were not significantly changed in FLC according to ChRO-Seq signal but were significantly reduced (gain of PTR [GPR]; *n* = 548) or increased (loss of PTR [LPR]; *n* = 240) according to RNA-Seq.

To determine whether one or more microRNAs serve as master regulators of the PTR genes, we employed miRhub (43), a bioinformatic tool that determines whether a set of genes is significantly enriched for predicted binding sites of any microRNA. We performed the analysis on GPR (*n* = 548) and LPR (*n* = 240) genes independently. Across both analyses, only 2 microRNAs emerged as candidate master microRNA regulators: miR-10b for GPR genes (Figure 7B) and miR-455 for LPR genes (Figure 7C).

### Inhibition of miR-10b reduces metabolic activity and proliferation in PDX-derived FLC cell lines.

Having determined that miR-10b is a candidate master regulator of GPR genes in FLC, we next sought to evaluate the potential function of miR-10b in FLC. We leveraged a primary human FLC cell line (designated FLC-C) that was established previously from a PDX (44) by treating with a locked nucleic acid (LNA) inhibitor against miR-10b. Following transfection, we assessed the impact on cell health and proliferation. We found that the number of metabolically active, viable cells was significantly reduced after miR-10b inhibition (Supplemental Figure 5, A and B).

**Figure 8:**
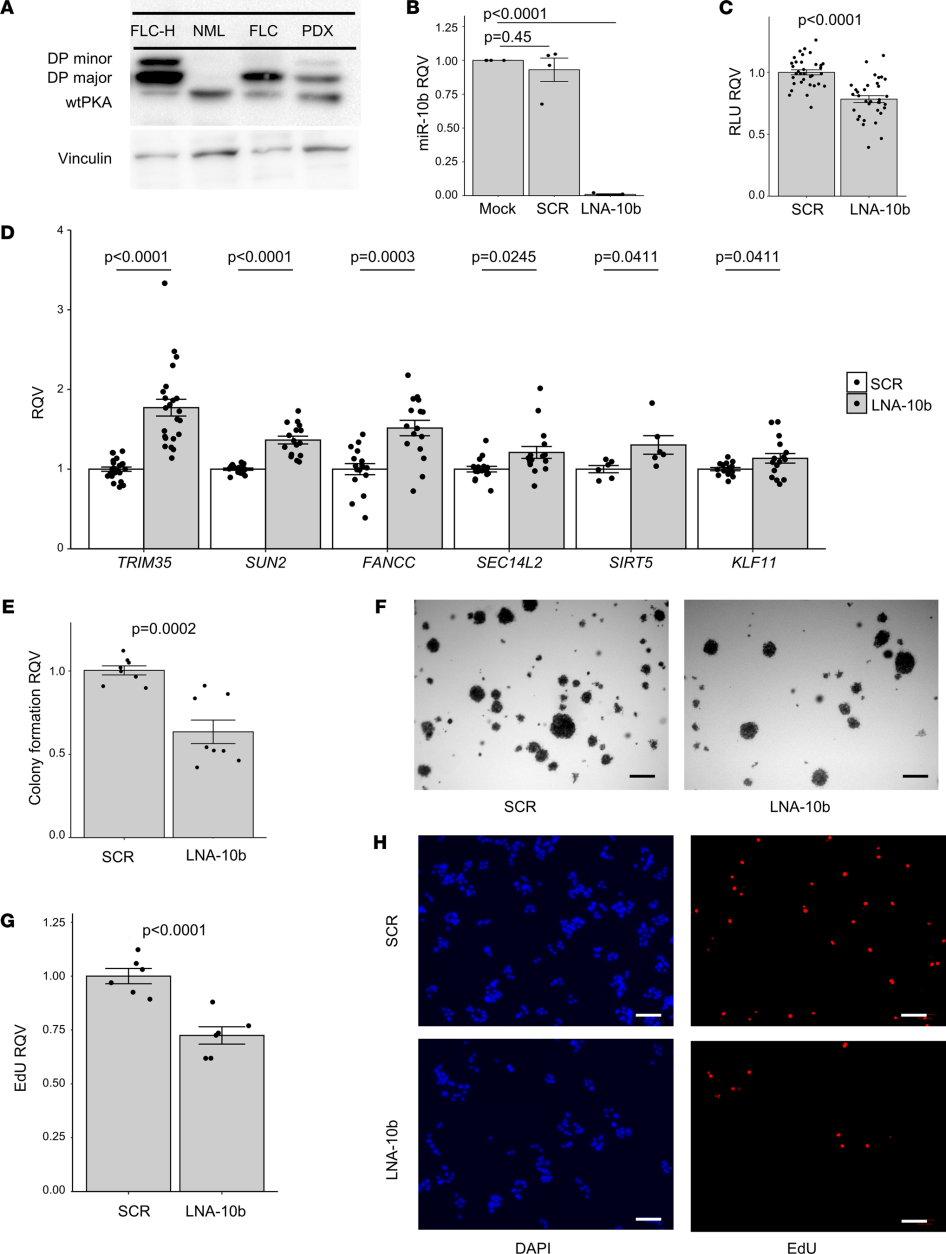
miR-10b inhibition reduces FLC cell metabolic activity and proliferation. (**A**) Protein expression of DNAJB1-PRKACA (DP) is detected with a protein kinase A catalytic α subunit (PKA) antibody. WT PKAc, DP major, and DP minor are identified. Lane 1, FLC-H cell line; lane 2, nonmalignant liver; lane 3, FLC patient sample; lane 4, FLC PDX sample. Vinculin expression for loading control is shown in the lower panel. Uncropped immunoblot shown in Supplemental Figure 4, A and B. (**B**) qPCR showing the RQV of miR-10b in FLC-H cells 6 days after 500 nM treatment with miR-10b LNA or scrambled sequence compared with mock (*n* = 4 each condition). (**C**) Luciferase signal (RLU) in FLC-H cells after 6 days of 500 nM miR-10b LNA treatment is shown as RQV compared with the scrambled negative control (6 trials, *n* = 6 each condition). (**D**) qPCR showing the RQV of *FANCC*, *KLF11*, *SEC14L2*, *SIRT5*, *SUN2*, and *TRIM35* in FLC-H cells after 6 days of 500 nM miR-10b LNA treatment compared with the negative control (*n* = 5–7 trials with 3 replicates for each condition, *SIRT5* *n* = 2 trials). (**E**) Soft agar colony formation of FLC-H cells 35 days after 500 nM miR-10b LNA compared with the negative control shown as RQV. (**F**) Representative nitro blue tetrazolium–stained images shown (2 trials, *n* = 8 each condition). (**G**) EdU incorporation in FLC-H cells 6 days after 500 nM treatment with miR-10b LNA compared with the negative control shown as RQV (2 trials, *n* = 6 each condition). (**H**) Representative DAPI- and EdU-stained images show total and proliferative cells, respectively. Scale bars: 100 μm. In all assays, each dot represents the average signal across technical replicates for a single biological replicate. *P* values are calculated by 2-tailed Student’s *t* test. *P* values reported in **B** and **D** were adjusted for multiple testing correction post hoc by the Benjamini-Hochberg method.

There are several limitations of the FLC-C line (21), including that it is viable for only very few passages; therefore, we pursued analysis of miR-10b function in an independently PDX-derived human FLC cell line (designated FLC-H). DP protein expression in FLC-H cells was similar to that of primary FLC tumors (Figure 8A and Supplemental Figure 4, A and B). The FLC-H cell line, and the PDX tumor from which it was derived, express both the major and minor isoform of DP, whereas the FLC tumor control used only expresses the major form of DP (10). We analyzed the expression of miR-10b by qPCR in FLC-H cells; although levels were slightly lower than in FLC tumor samples (Supplemental Figure 5C), they were highly stable across multiple passages (Supplemental Figure 5D). With this improved cell line, we confirmed that miR-10b abundance was dramatically reduced after transfection with the LNA inhibitor (Figure 8B). We then confirmed that miR-10b reduction led to modest (~25%) but significant reduction in metabolically active, viable cells as measured by CellTiter-Glo (*n* = 6 independent experiments representing 34 biological replicates, Figure 8C).

Predicted targets of miR-10b among the GPR genes included Fanconi Anemia Complement Group C (*FANCC*), Kruppel Like Factor 11 (*KLF11*), SEC14 Like Lipid Binding 2 (*SEC14L2*), Sirtuin 5 (*SIRT5*), Sad1 and UNC84 Domain Containing 2 (*SUN2*), and Tripartite Motif Containing 35 (*TRIM35*), all of which have been implicated previously in the control of cell proliferation (25, 28, 31, 45–48). We measured the expression level of these genes by qPCR after LNA-mediated miR-10b inhibition and observed small but significant changes in *KLF11, SIRT5,* and *SEC14L2*; a modest and significant upregulation of *FANCC* and *SUN2*; and a robust and significant increase in expression of *TRIM35* (~2-fold) (Figure 8D). We then measured the expression of these genes after transfection of a miR-10b mimic (MIM), which dramatically increased the abundance of miR-10b (Supplemental Figure 5E). We first measured previously reported targets of miR-10b (37), *CDH1* and *PTEN*, and found that neither LNA-10b nor MIM-10b altered the expression of these genes (Supplemental Figure 5F). We did, however, observe a modest reduction of *SIRT5*, *SUN2*, and *TRIM35* and a significant reduction of *FANCC* mRNA after MIM-10b treatment (Supplemental Figure 5G). Based on the known functions of FANCC, TRIM35, and SUN2, we hypothesized that the reduction in viable cells after miR-10b inhibition was due to reduced proliferation. To test this, we performed an anchorage-independent growth assay and an EdU incorporation assay (to assess replication and synthesis), and we also carried out TUNEL staining (to assess apoptotic induction) in the FLC-H cell line. Inhibition of miR-10b led to a significant reduction in colony growth (~40%) (Figure 8, E and F) and EdU incorporation (~25%) (Figure 8, G and H), but it led to no change in TUNEL staining (Supplemental Figure 5, H and I). Taken together, these results indicate that miR-10b regulates cell proliferation but not apoptosis in FLC cells.

## Discussion

FLC is an aggressive cancer afflicting young adults who lack an underlying predisposition to liver cancer. Surgical resection is the primary remedy for FLC, but advanced disease is incurable, as there are no standard-of-care therapeutics. While it is well established that patients with FLC harbor the oncogene *DNAJB1-PRKACA*, a direct and specific inhibitor of the DP enzyme is unavailable. Furthermore, it remains unclear whether DP is essential for tumor progression, maintenance, and metastasis. For these reasons, there is a dire need to identify alternative therapeutic strategies. Toward that end, it is important first to deepen understanding of the molecular underpinnings of FLC etiology and progression. In this study, we focused our investigation on the contributions of posttranscriptional gene regulation and microRNA activity toward shaping gene expression profiles and tumor phenotypes of FLC.

We first performed microRNA analysis in the largest FLC and NML sample set to date — more than 3 times as large as our previous study, which centered on the potential role of miR-375 as a tumor suppressor in FLC (22). The sample set used in the present study enabled analyses that were infeasible in previous studies of microRNAs in FLC (22, 23). Specifically, we were able to compare matched samples from the same patient and also directly compare primary versus metastatic FLC. Moreover, we were also able to assess the potential effect of metastatic location on the microRNA landscape.

A major strength of the study is the inclusion of ChRO-Seq data to quantify the rates of transcription for microRNAs that we found to be differentially expressed in FLC. In most cases, the change in transcription at a microRNA locus (as determined by ChRO-Seq) matched the change in expression of the mature microRNA (as determined by small RNA-Seq). However, there were some notable exceptions, such as miR-203 and miR–378a-5p. For these microRNAs, the amplitude of the fold-change in expression was much greater than the fold-change in transcription, suggesting that they may be altered in FLC primarily by posttranscriptional mechanisms.

Another new aspect of the current study is the integrative analysis of ChRO-Seq and RNA-Seq data to identify a high-confidence list of genes that are subject primarily to PTR in FLC. Specifically, we defined those genes that change significantly in expression in FLC due primarily to GPR or LPR. GPR and LPR genes represent the top candidate targets of microRNAs and RNA binding proteins (RBPs), which are the major mechanisms of posttranscriptional gene expression control. In this study, we focused on microRNAs, but the contribution of RBPs merits investigation in the future. We found that miR-10b is the top candidate master regulator of GPR genes and miR–455-3p is the top candidate master regulator of LPR genes.

Although we found that several microRNAs were altered in FLC, we decided to pursue miR-10b for functional studies for 3 major reasons brought to light by our study: (a) it was the most highly expressed among the upregulated microRNAs in FLC; (b) it was the most transcriptionally activated microRNA in FLC compared with NML; and (c) it was the only upregulated microRNA in FLC that was specifically and robustly responsive to DP activity (and not just WT PKA activity) in 2 different human cell models (HepG2 and HEK293). Interestingly, miR-10b levels were not increased by the presence of DP in 2 different mouse cell models or the only available in vivo mouse model, which suggests that the regulatory connection between DP and miR-10b is species-specific. It is important to note here the limitation that HepG2 and HEK293 cell models do not replicate the environment in which FLC tumors are initiated in humans, though the data from the analysis of the tissue from the patient with IOPN does provide in vivo support for a regulatory connection between DP and miR-10b. The FLC cell of origin has not yet been determined, though at least 1 study has suggested biliary tree stem cells as a possibility (44). In the future, it will be important to assess whether miR-10b and other FLC markers and candidate drivers are sensitive to DP activity in biliary tree stem cells or other candidate cells of origin.

In other cancer types, the transcription factor TWIST1 promotes miR-10b expression (49, 50), and the activation of TWIST1 protein by phosphorylation is PKA dependent (51, 52). Considering that DP is a chimera that maintains PKA catalytic activity, a similar mechanism may operate in FLC. TWIST1 activity is also enhanced by the transcription factor FOXQ1 (53), and we predicted several high-confidence FOXQ1 binding sites in the promoter of miR-10b in FLC. FOXQ1 promotes PI3K/AKT activity in colorectal cancer (54), and the abundant expression of FOXQ1 in FLC suggests that a similar mechanism may exist. The potential role of FOXQ1 in promoting miR-10b expression in FLC, with or without TWIST1, is intriguing and merits further investigation. MiR-10b has been shown to contribute to the progression of 15 different cancer types (37), generally by promoting cancer cell metastasis. In our experiments, we found that the upregulation of miR-10b in FLC was greater than in any other cancer type for which data are available in TCGA. Our finding that *TRIM35* was regulated by miR-10b is potentially novel. In hepatocellular carcinoma, TRIM35 has been identified as a tumor suppressor (31) by limiting the stability of pyruvate kinase and reducing cellular energetics. In future studies, it will be interesting to evaluate whether miR-10b controls glycolysis in FLC cells and whether this regulation is mediated by suppression of TRIM35. MiR-10b regulation of SUN2 is also potentially new. The reported role of SUN2 in suppressing the Warburg effect (25) provides another clue about the possible role of miR-10b in promoting glycolysis in FLC cells.

The functional experiments in this study reveal that suppression of miR-10b in FLC cells led to a modest but significant reduction in metabolic activity, anchorage-independent cell growth, and proliferation. These results point to a role for miR-10b in promoting the growth of FLC cells. Due to the modest effects of miR-10b on growth/proliferation, we do not propose that a miR-10b inhibitor is, by itself, a candidate therapeutic modality. However, we do believe that the functions of miR-10b in FLC are worth studying further in improved models of FLC (e.g., additional PDX models or direct patient-derived cells) and that, in the future, it may be worth considering a miR-10b inhibitor as part of a combinatorial therapeutic approach. Interestingly, many genes established as miR-10b targets previously in other cancer types, such as *PTEN* (55), were not identified as significantly altered in FLC, which speaks to the growing appreciation for contextual assessment of microRNA targets. Also, although we did not functionally interrogate miR–455-3p in this study, we believe it merits consideration in the future, especially since it has been reported already as a key suppressor of invasion and metastasis in other aggressive cancers, particularly esophageal squamous cell carcinoma, colorectal cancer, breast cancer, melanoma, and pancreatic cancer (56–59).

## Methods

### Human samples.

Informed consent was obtained from all individuals. Tumor and adjacent NML samples were collected from patients with FLC by surgeons and provided by the FCF. Patients were males and females, and some samples were collected from the same patient. All samples were deidentified before shipment to Cornell. Additional sample information is detailed in Supplemental Table 1.

The tissue sample from the patient with IOPN, as well as the adjacent normal pancreas tissue sample, was obtained from Olca Basturk at the Memorial Sloan Kettering Cancer Center (New York, New York, USA).

### Animal samples.

Liver samples from female C57BL/6N mice on 3,5-diethoxycarbonyl-1,4-dihydrocollidine (DDC, 0.1%) diet were obtained from a previous study (14). These liver samples expressed human *DNAJB1-PRKACA*, β-catenin, both DNAJB1-PRKACA and β-catenin, or an empty vector control and were collected 4.5 months after transposition of *DNAJB1-PRKACA*.

### Cell lines.

HepG2 cells were obtained from the American Type Culture Collection (ATCC). HepG2 cells expressing *GFP*, *DNAJB1-PRKACA*, *PRKACA*, or the *DNAJB1-PRKACA* K128H mutant have been previously described (21, 22). HepG2 cells were cultured in DMEM containing 1 g/L glucose (Thermo Fisher Scientific) supplemented with 10% FBS (Thermo Fisher Scientific), 1% GlutaMAX (Thermo Fisher Scientific), 110 mg/L sodium pyruvate (Thermo Fisher Scientific), 1% penicillin-streptomycin (Thermo Fisher Scientific), and puromycin 5 μg/mL (Thermo Fisher Scientific).

AML-12 cells expressing *DNAJB1-PRKACA* have been previously described (42) and were grown in DMEM:F12 medium (Thermo Fisher Scientific) supplemented with 10% FBS (Thermo Fisher Scientific), 10 μg/mL insulin (Thermo Fisher Scientific), 5.5 μg/mL transferrin (Thermo Fisher Scientific), 5 ng/mL selenium (Thermo Fisher Scientific), and 40 ng/mL dexamethasone (Thermo Fisher Scientific).

HEK293 cells expressing *DNAJB1-PRKACA* have been previously described (40) and were grown in DMEM containing 4.5 g/L glucose (Thermo Fisher Scientific) supplemented with 10% FBS (Thermo Fisher Scientific), 1% GlutaMAX (Thermo Fisher Scientific), 110 mg/L sodium pyruvate (Thermo Fisher Scientific), and 1% penicillin-streptomycin (Thermo Fisher Scientific).

TIB75 cells expressing *DNAJB1-PRKACA* were obtained as a gift from Mark Yarchoan (Johns Hopkins, Baltimore, Maryland, USA). TIB75 cells are derived from embryonic murine liver epithelia then transformed with methylcholanthrene epoxide and made available through the ATCC. Cells were treated with a CRISPR/Cas9 guide and selected as previously described (42). These cells were grown in DMEM containing 4.5 g/L glucose (Thermo Fisher Scientific) supplemented with 10% FBS (Thermo Fisher Scientific), 1% GlutaMAX (Thermo Fisher Scientific), 110 mg/L sodium pyruvate (Thermo Fisher Scientific), and 1% penicillin-streptomycin (Thermo Fisher Scientific).

FLC-C cells were generated from a PDX model (44) and grown in advanced DMEM media conditioned by irradiated mouse embryonic fibroblasts containing 300 mg/L L-glutamine (Thermo Fisher Scientific) supplemented with 10% FBS (Thermo Fisher Scientific) and 1% penicillin-streptomycin (Thermo Fisher Scientific). The media was supplemented with 10% DMEM from cultured human embryonic kidney cells harboring a human RSPO1 transgene and 20 μM Y-27632 ROCK inhibitor as previously described (21).

FLC-H cells were generated from a PDX model (44) and grown in RPMI1640 media containing 300 mg/L L-glutamine (Thermo Fisher Scientific) supplemented with 10% FBS (Thermo Fisher Scientific), 1% penicillin-streptomycin (Thermo Fisher Scientific), and 2.5 μg/mL human hepatic growth factor (Thermo Fisher Scientific).

All cell lines were cultured in a humid chamber at 37°C and 5% CO_2_.

### Small RNA library preparation and sequencing.

Frozen tumors underwent physical dissociation using a polytron PT1200 E homogenizer (Thomas Scientific), and total RNA was isolated with the Total RNA Purification Kit (Norgen Biotek) as per the manufacturer’s instructions. RNA purity was quantified with the Nanodrop 2000 instrument (Thermo Fisher Scientific), and RNA integrity was quantified with the Agilent 4200 Tapestation (Aglient Technologies). Libraries were generated using the CleanTag Small RNA Library Prep Kit (TriLink Biotechnologies). Sequencing was performed on the HiSeq2000 or HiSeq3000 platforms (Illumina) at the Genome Sequencing Facility of the Greehey Children’s Cancer Research Institute (University of Texas Health Science Center). RNA from cell lines was purified and sequenced as described above except without polytron homogenization.

### PolyA+ RNA library preparation and sequencing.

Of the 27 RNA-Seq data sets analyzed in this study, 18 were generated and published previously (21), and 9 were newly generated. For the newly generated data, total RNA was isolated using the Total RNA Purification Kit (Norgen Biotek) per manufacturer’s instructions. RNA purity was quantified with the Nanodrop 2000 (Thermo Fisher Scientific) or Nanodrop One, and RNA integrity was quantified with the Agilent 4200 Tapestation (Agilent Technologies). Libraries were prepared by the Cornell Transcriptional Regulation and Expression (TREx) Facility using the NEBNext Ultra II Directional RNA kit. Sequencing was performed at the Biotechnology Research Center at Cornell University on the NextSeq500 (Illumina).

### Chromatin run-on library preparation and sequencing.

All ChRO-Seq data sets (*n* = 13) analyzed in this study were generated and published previously (21).

### Bioinformatic analysis.

Small RNA-Seq was processed using miRquant 2.0 as previously described (33). In brief, miRquant 2.0 trimmed the 3′ adapter from small RNA reads, aligned reads to the genome (hg19), annotated miRNAs (miRbase v18), identified deviations from canonical miRNAs (iso-miRNAs, nontemplate additions, internal edits), and quantified aligned reads as both raw counts and normalized reads per million mapped to miRNAs (RPMMM). Raw counts were analyzed using DESeq2.0 (v1.3) to obtain normalized counts and to determine differential expression, using a model that includes sequencing batch as a covariate. For visualization and clustering, normalized counts were transformed using a variance stabilizing transformation and corrected for batch effect using the removeBatchEffect function from the limma package.

Normalized small RNA-Seq data available from TCGA were acquired using TCGA-assembler 2, and comparative analysis was performed as previously described (22).

Paired-end RNA-Seq reads were aligned to the human genome (hg38) using STAR (v2.4.2a), and reads aligning to the transcriptome were quantified using Salmon (v0.6). Differential expression was determined with DESeq2.0 (v1.3) using a model that accounts for sequencing facility as a covariate.

miRhub (43) was used to determine if putative microRNA binding sites were enriched in a list of differentially expressed genes. In brief, miRhub uses a gene list, usually up- or downregulated genes, as an input. For each microRNA, the cumulative number of putative microRNA binding sites in the 3′ UTR of those genes represents the score for that microRNA. Using a Monte-Carlo simulation, this process was repeated 1000 times using random gene lists of the same length. The score from the input gene list was compared with scores of the simulated gene lists to determine significance.

### Quantification of transcriptional activity at microRNA loci.

ChRO-Seq data were published previously (21). MicroRNA loci were defined as the beginning of the nearest upstream transcriptional regulatory element (TRE), defined as the promoter, to the end of the mature microRNA. Total ChRO-Seq signal was calculated in this region as a measure of transcriptional activity. Genomic loci snapshots were generated using Gviz 1.26.5.

### Integration of RNA-Seq and ChRO-Seq data to identify genes subject primarily to PTR.

We first performed a differential transcription analysis of gene bodies and differential expression analysis of genes independently using the DESeq2.0 (v1.3). Genes gaining PTR were identified as those not altered at the transcriptional level between FLC and NML (ChRO-Seq log_2_FC > –0.59 and ChRO-Seq FDR > 0.2), but significantly downregulated at the steady state gene expression level (RNA-Seq average NML counts > 1000, RNA-Seq log_2_FC < –1, and RNA-Seq FDR < 0.05). Genes losing PTR were identified as those not altered at the transcriptional level between FLC and NML (ChRO-Seq log_2_FC < 0.59 and ChRO-Seq FDR > 0.2), but they were significantly upregulated at the steady state gene expression level (RNA-Seq average FLC counts > 1000, RNA-Seq log_2_FC > 1, and RNA-Seq FDR < 0.05). We determined using miRhub if predicted targets of any of the FLC-dysregulated microRNAs were significantly enriched in these lists of posttranscriptionally regulated genes.

### qPCR.

Total RNA was isolated from tissue or cells using Total RNA Purification Kit (Norgen Biotek) as per manufacturer’s instructions. Reverse transcription was performed using the High-Capacity RNA-to-cDNA Kit (Thermo Fisher Scientific) for gene analysis or using the TaqMan MircoRNA Reverse Transcription Kit (Thermo Fisher Scientific) for microRNA analysis. Gene and microRNA expression were quantified with TaqMan Expression assays on a CFX96 Touch Real-Time System thermocycler (Bio-Rad). Gene expression assays were normalized to the expression of *RPS9*, and microRNA expression assays were normalized to the expression of *RNU6*. Individual gene assay IDs include the following: CDH1, hs01023895; DNAJB1-PRKACA, custom; FANCC, hs0098454; KLF11, hs00231614; miR-10b, 002218; miR-21, 000397; miR-182, 002334; PTEN, hs02621230; RNU6, 001973; RPS9, hs02339424; SEC14L2, hs00391446; SIRT5, hs00978331; SUN2, hs00391446; and TRIM35, hs00324633 (Thermo Fisher Scientific). Expression values reported are averaged across at least 3 biological replicates unless otherwise stated in the main text.

### Immunoblot analysis.

HepG2 and FLC-H cells were lysed in RIPA buffer containing Halt protease and phosphatase inhibitors (Thermo Fisher Scientific) at 4°C. Cells were incubated for 30 minutes and centrifuged at 14,000*g* for 10 minutes at 4°C. Total protein in the supernatant was quantified using the BCA Protein Assay Kit (Thermo Fisher Scientific). Samples were denatured in NuPAGE LDS Sample Buffer (Thermo Fisher Scientific) containing 5% β-Mercaptoethanol for 10 minutes at 70°C and loaded to a 12% SDS-polyacrylamide gel. After electrophoresis, samples were transferred to polyvinylidene difluoride membrane and blocked in Tris buffered saline containing 0.5% TWEEN20 (TBST) and 3% BSA for 1 hour at room temperature. Membranes were probed for anti-PRKACA (1:1000 dilution, rabbit source, Cell Signaling Technology, 4782) or anti-Vinculin (1:1000 dilution, Invitrogen, MA5-11690) overnight at 4°C and then incubated with goat anti–rabbit HRP linked IgG (1:10000, Cell Signaling Technology). Membranes were visualized using a ChemiDoc MP (Bio-Rad).

### Cell count, alamarBlue, Cell Titer Glo, EdU incorporation, soft agar, and TUNEL assays.

HepG2 cell lines were plated at a density of 10,000 cells/well in 6-well plates. Cells were collected daily for 5 days, stained with trypan blue, and counted with a TC20 automated cell counter (Bio-Rad). Six independent counts were performed, and the assay was repeated twice.

FLC-H cells were plated at a density of 10,000 cells/well in 96-well plates. After overnight incubation, cells were transfected with miR-10b LNA inhibitor or scramble negative control (Qiagen) at a 500 nmol/L final concentration using Lipofectamine 3000 (Thermo Fisher Scientific) according to manufacturer’s instructions. Six days after transfection, cells were assayed for health using alamarBlue or the Cell Titer Glo Kit (Promega). To determine the metabolic health of cells, alamarBlue was added to cells, per the manufacturer’s instruction, and they were incubated for 3 hours and assayed for spectral absorbance at 570 nm. The Cell Titer Glo assay was used to determine the amount of ATP as relative fluorescent units (RFU) of luciferase activity. For each experiment, the signal across 6 wells was averaged and normalized to the scramble LNA-treated cells.

To quantify anchorage-independent growth, FLC-H cells were plated at a density of 10,000 cells/well in 6-well plates. Cells are mixed with media containing 0.3% agar and plated on a prehardened layer of media containing 0.6% agar. Both layers of media contain LNA at a concentration of 500 nmol/L. Cells were incubated for 35 days and were provided supplemental media containing 0.3% agar and LNA to prevent drying. At the end of the growth period, 200 μL of a 1 mg/mL solution of nitro blue tetrazolium chloride was added to each well and incubated overnight at 37°C. Eight biological replicates were performed, and at least 10 independent fields per well were imaged. Images were manually analyzed in ImageJ (NIH), and data are represented as the percent area stained.

To quantify the proliferation rate, 6 days after transfection, the cells were treated for 2 hours with 10 μmol/L EdU. EdU-labeled cells were washed with phosphate-buffered saline, fixed in 4% paraformaldehyde for 20 minutes, and permeabilized with 0.5% Triton-x 100 for 20 minutes. EdU was detected using the Click-iT Plus EdU Alexa Fluor 594 Imaging kit (Thermo Fisher Scientific) according to the manufacturer’s instructions, with reaction volumes appropriately scaled for 96-well plates.

To quantify apoptosis, 6 days after transfection, the cells were washed with phosphate-buffered saline, fixed in 4% paraformaldehyde for 20 minutes, and permeabilized with 0.5% Triton-x 100 for 20 minutes. Apoptotic cells were detected using the Click-iT TUNEL Alexa Fluor 488 Imaging kit (Thermo Fisher Scientific) according to the manufacturer’s instructions, with reaction volumes appropriately scaled for 96-well plates.

After EdU and TUNEL detection, the cells were counterstained with DAPI. Images were collected on a ZOE Fluorescent Cell Imager (Bio-Rad). For each experiment, 4 independent fields per well were imaged. Images were manually analyzed in ImageJ. EdU^+^ and TUNEL^+^ cells are represented as a ratio of all cells in each field, which were averaged and normalized to the scramble treated cells. Each assay was performed with 6 biological replicates unless otherwise stated in the text.

### Data availability.

All unpublished small RNA-Seq and RNA-Seq data can be downloaded from Gene Expression Omnibus (GEO) using accession no. GSE181922. Previously published small RNA-Seq can be downloaded from GEO using accession no. GSE114974. Previously published RNA-Seq and ChRO-Seq can be downloaded from the European Genome-Phenome Archive (EGA) using the following EGA accession number: EGAS00001004169.

### Statistics.

Statistical comparisons of qPCR, alamarBlue, Cell Titer Glo, EdU, and TUNEL results were made using Student’s 2-tailed *t* test. FDR was controlled for by applying Benjamini-Hochberg correction to experiments where multiple comparisons were made. Significant differences in gene expression or transcriptional signal were determined using DESeq2.0. Graphs were generated in the R software package, and data are shown as mean ± SEM.

### Study approval.

Informed consent was obtained from all individuals involved in this study and approved by the IRB protocols 1802007780, 1811008421 (Cornell University), and 33970/1 (FCF). Animal samples used in this study were supplied by Scott Lowe at the Memorial Sloan Kettering Cancer Center and approved by the IACUC protocol 11-06-011 (the Memorial Sloan Kettering Cancer Center).

## Author contributions

ABF designed the study; acquired, analyzed, and interpreted data; prepared figures; provided statistical analysis; and drafted the manuscript. MK analyzed and interpreted data, prepared figures, provided statistical analysis, and edited the manuscript. APM acquired, analyzed, and interpreted data and edited the manuscript. TAD and RS provided material support and edited the manuscript. KV and NB obtained funding and provided resources. PS designed the study, analyzed and interpreted data, drafted the manuscript, obtained funding, and supervised the study.

## Supplementary Material

Supplemental data

Supplemental table 1

Supplemental table 2

## Version 1 04/28/2022

In-Press Preview

## Version 2 06/08/2022

Electronic publication
