# Supplementary material for: Multiomic analysis of microRNA-mediated regulation reveals a proliferative axis involving miR-10b in fibrolamellar carcinoma
Source: JCI Insight. 2022 Jun 8;7(11):e154743. doi: 10.1172/jci.insight.154743 (PMC9220943; doi:10.1172/jci.insight.154743)
Supplement: Supplemental data [file jciinsight-7-154743-s130.pdf]

Supplemental Figure 1

A

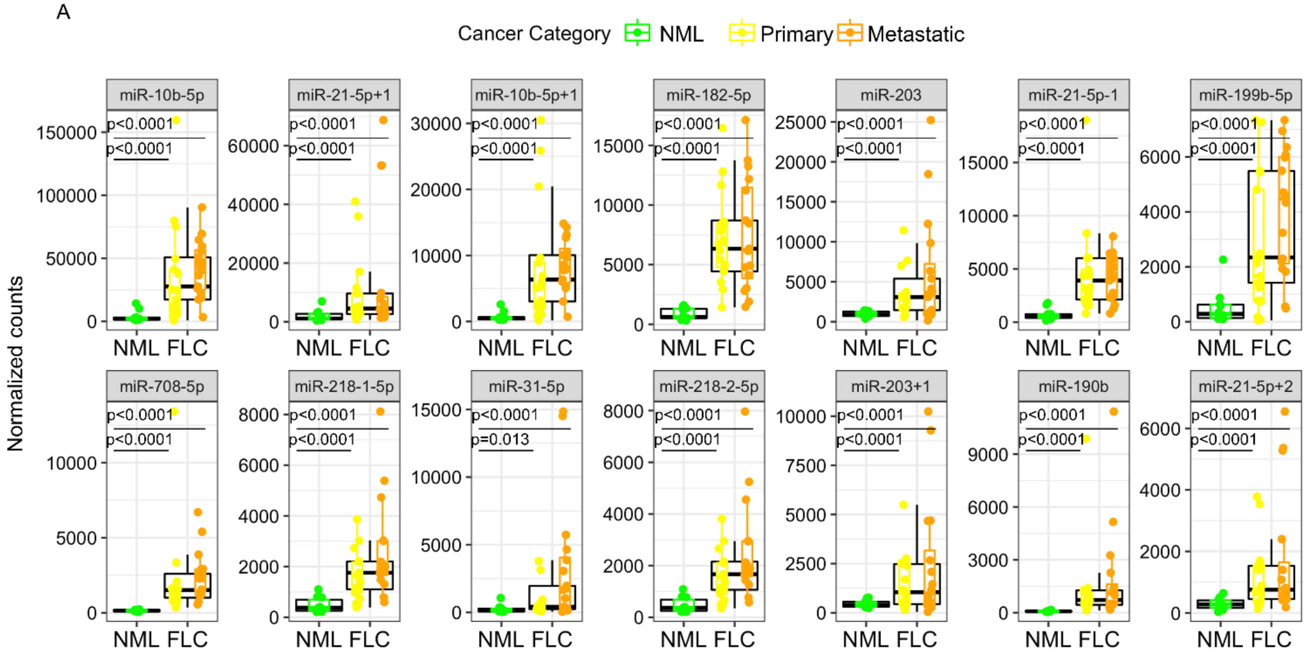

Supplemental Figure 1. Expression of the up-regulated microRNAs in primary and metastatic FLC patient samples. The normalized read counts of each up-regulated microRNA in either primary or metastatic. Each data point represents a patient sample. *P*-values are calculated by two-tailed Student's t-test.

Supplemental Figure 2

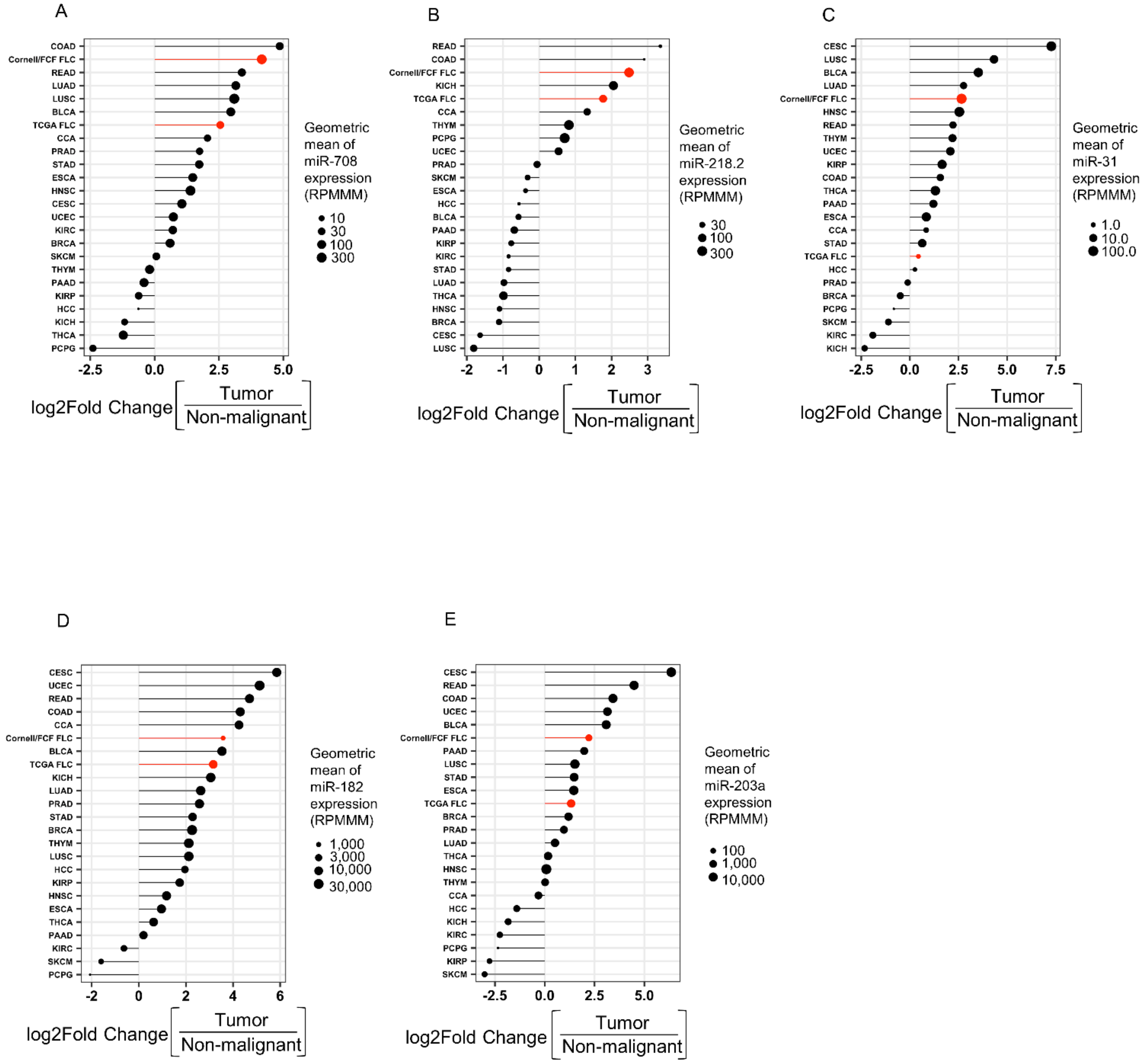

Supplemental Figure 2. FLC microRNA expression compared to other cancer types. (A-E) Log2 FC expression of the 5 next most up-regulated microRNAs in FLC (after removing isomiRs) within TCGA. The size of each circle represents the geometric mean of microRNA expression in each tumor type. Each tumor type is ranked on the y-axis by the log2 FC of the geometric mean of tumor expression relative to non-tumor expression. The FLC sample set used in this study (Cornell/FCF) and the FLC sample set available from TCGA (n=6) are highlighted in red. BLCA, bladder urothelial carcinoma; BRCA, breast invasive carcinoma; CESC, cervical squamous cell and endocervical adenocarcinoma; CCA, cholangiocarcinoma; COAD, colon adenocarcinoma; Cornell/FCF FCL, fibrolamellar carcinoma samples analyzed in this study; ESCA, esophageal carcinoma; HCC, hepatocellular carcinoma; HNSC, head and neck squamous cell carcinoma; KICH, kidney chromophobe; KIRC, kidney renal papillary cell carcinoma; KIRP, kidney renal clear cell carcinoma; LUAD, lung adenocarcinoma; LUSC, lung squamous cell carcinoma, PAAD, pancreatic adenocarcinoma; PCPG, pheochromocytoma and paraganglioma; PRAD, prostate adenocarcinoma; READ, rectum adenocarcinoma; SKCM, skin cutaneous melanoma; STAD, stomach adenocarcinoma; TCGA FLC; fibrolamellar carcinoma; THCA, thyroid carcinoma; THYM, thymoma; UCEC, uterine corpus endometrial carcinoma; RPMMM = Reads per million mapped to microRNAs.

Supplemental Figure 3

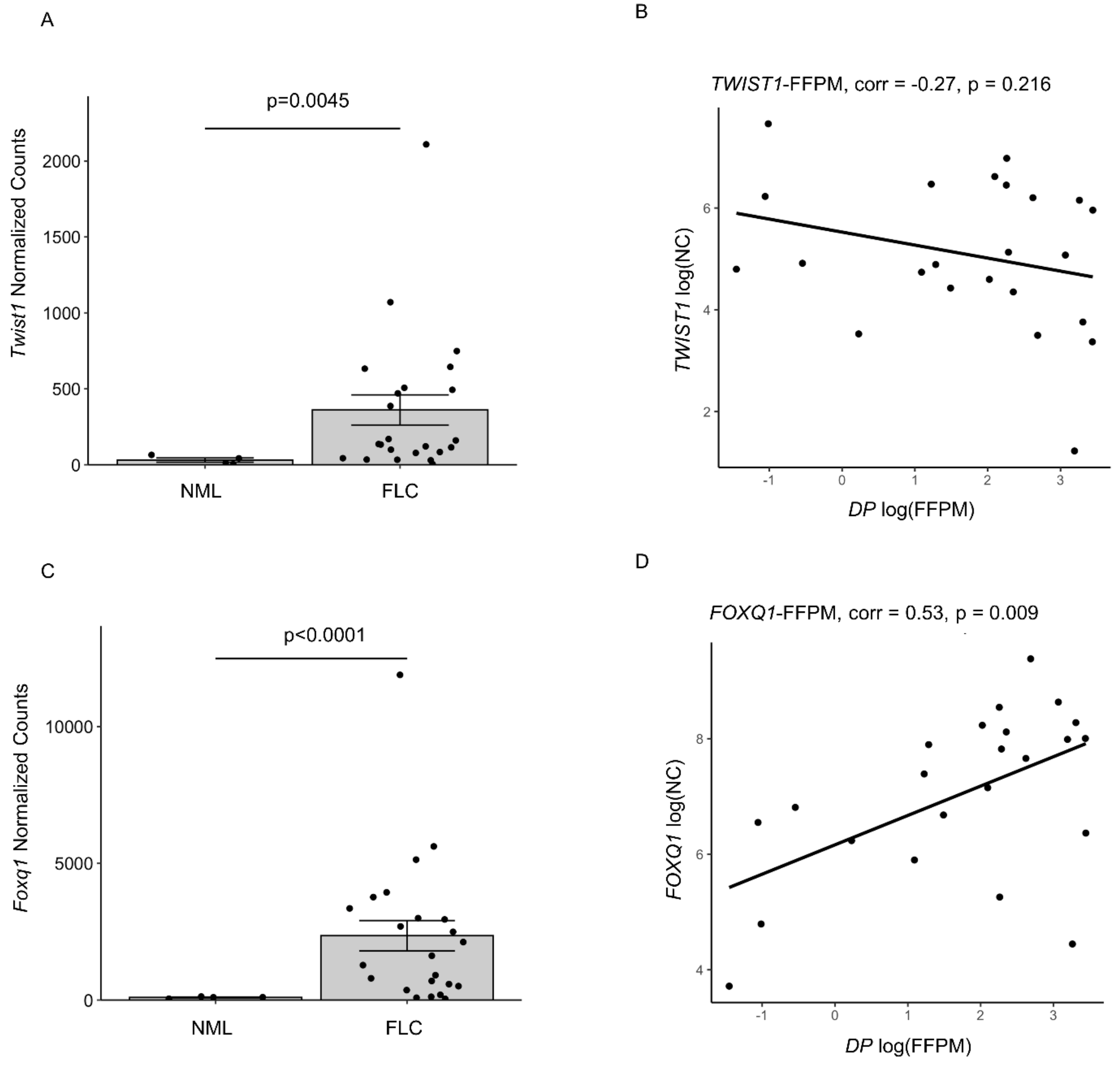

Supplemental Figure 3. FOXQ1 mRNA levels correlates to DP expression. (A) Bar graph showing the normalized RNA-seq read counts for *TWIST1* in NML and FLC. **(B)** Correlation of *TWIST1* (y-axis) and *DP* (x-axis). **(C)** Bar graph showing the normalized RNA-seq normalized read counts for *FOXQ1* in NML and FLC. **(D)** Correlation of *FOXQ1* (y-axis) and *DP* (x-axis). Expression shown as the log of normalized counts in FLC samples with RNA-seq data (n=19). Measurements from individual samples are shown as data points. *P*-values calculated by two-tailed Student's t-test.

Supplemental Figure 4

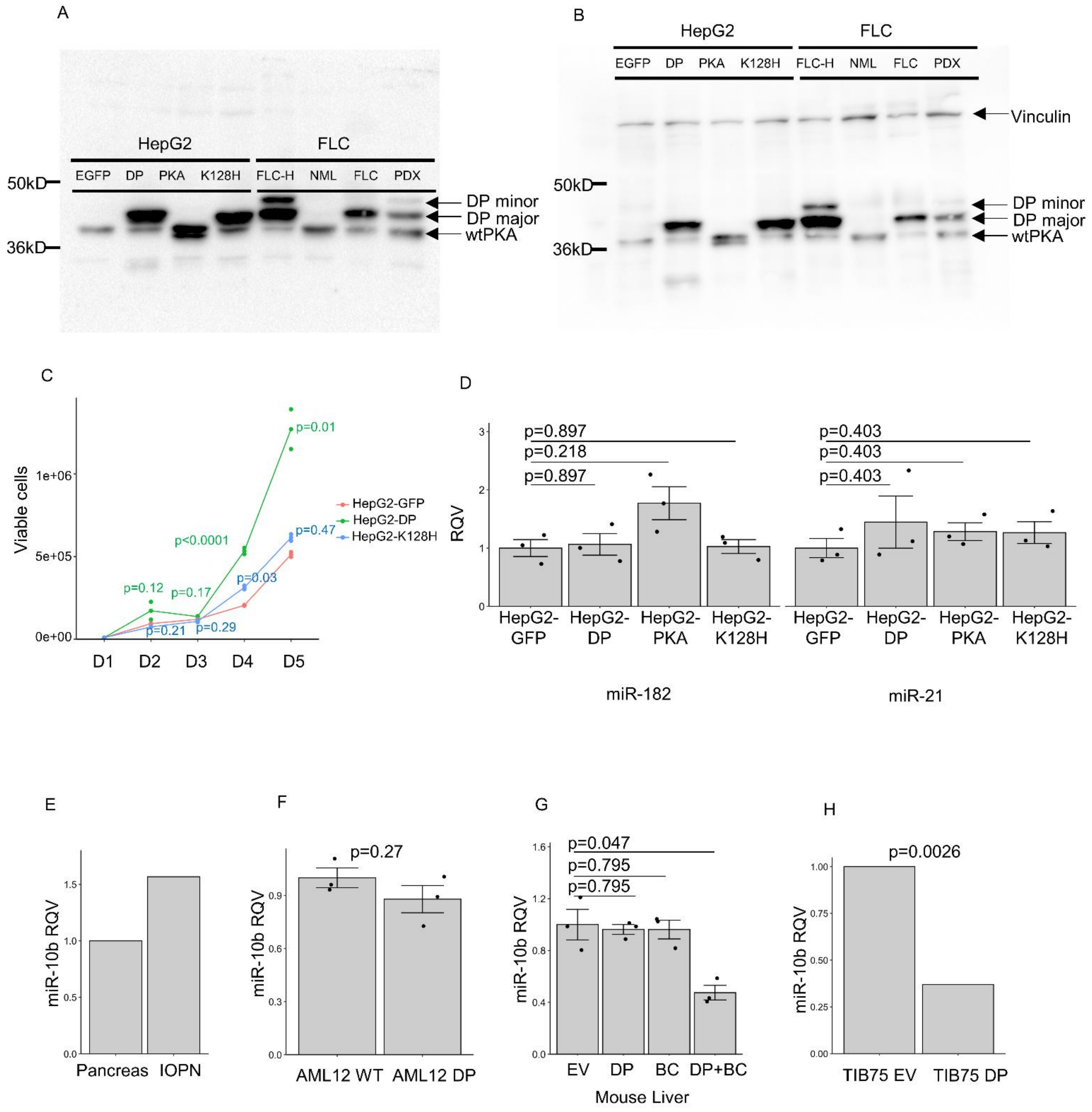

Supplemental Figure 4. (A) Immunoblot probed with a PKAc antibody. Wild-type PKAc and DP are identified. Lane 1, HepG2-GFP; lane 2, HepG2-DP; lane 3, HepG2-PKA; lane 4, HepG2-K128H; lane 5, FLC-H cell line; lane 6, non-malignant liver (NML) sample; lane 7, Fibrolamellar carcinoma (FLC) sample; lane 8, patient-derived xenograft (PDX) sample. Molecular masses from the ladder of 36 and 50 kilodaltons are denoted. **(B)** Immunoblot from panel A probed with vinculin antibody. Disclaimer: cropped content of immunoblot is shown in Figure 6B and Figure 8A. **(C)** Growth of HepG2-GFP, HepG2-DP, and HepG2-K128H cell lines over 5 days (n=3 each line). **(D)** Quantitative PCR showing the RQV of miR-10b in an intraductal oncocytic papillary neoplasm (IOPN) sample relative to healthy pancreatic tissue (Pancreas) (n=1). **(E)** Quantitative PCR showing the RQV of miR-182 and miR-21 expression in HepG2-GFP, HepG2-DP, HepG2-PKA, and HepG2-K128H cell lines (n=3 each line). **(F)** Quantitative PCR showing the RQV of miR-10b in AML12 wild type (WT) and DNAJB1-PRKACA over-expression (DP) cell lines (n=3 each line). **(G)** Quantitative PCR showing the RQV of miR-10b in mouse livers transposed with empty vector (EV), DNAJB1-PRKACA expression (DP), constitutively active β-catenin expression (BC), or DNAJB1-PRKACA and constitutively active β-catenin expression (DP+BC) (n=3 each condition). **(H)** Quantitative PCR showing the RQV of miR-10b in TIB75 empty vector (EV) and DNAJB1-PRKACA over-expression (DP) cell lines. In all assays, each dot represents the average signal across technical replicates for a single biological replicate. *P*-values calculated by two-tailed Student's t-test. *P*-values reported in panels D and G were adjusted for multiple testing correction post-hoc by benjamini-hochberg method.

Supplemental Figure 5

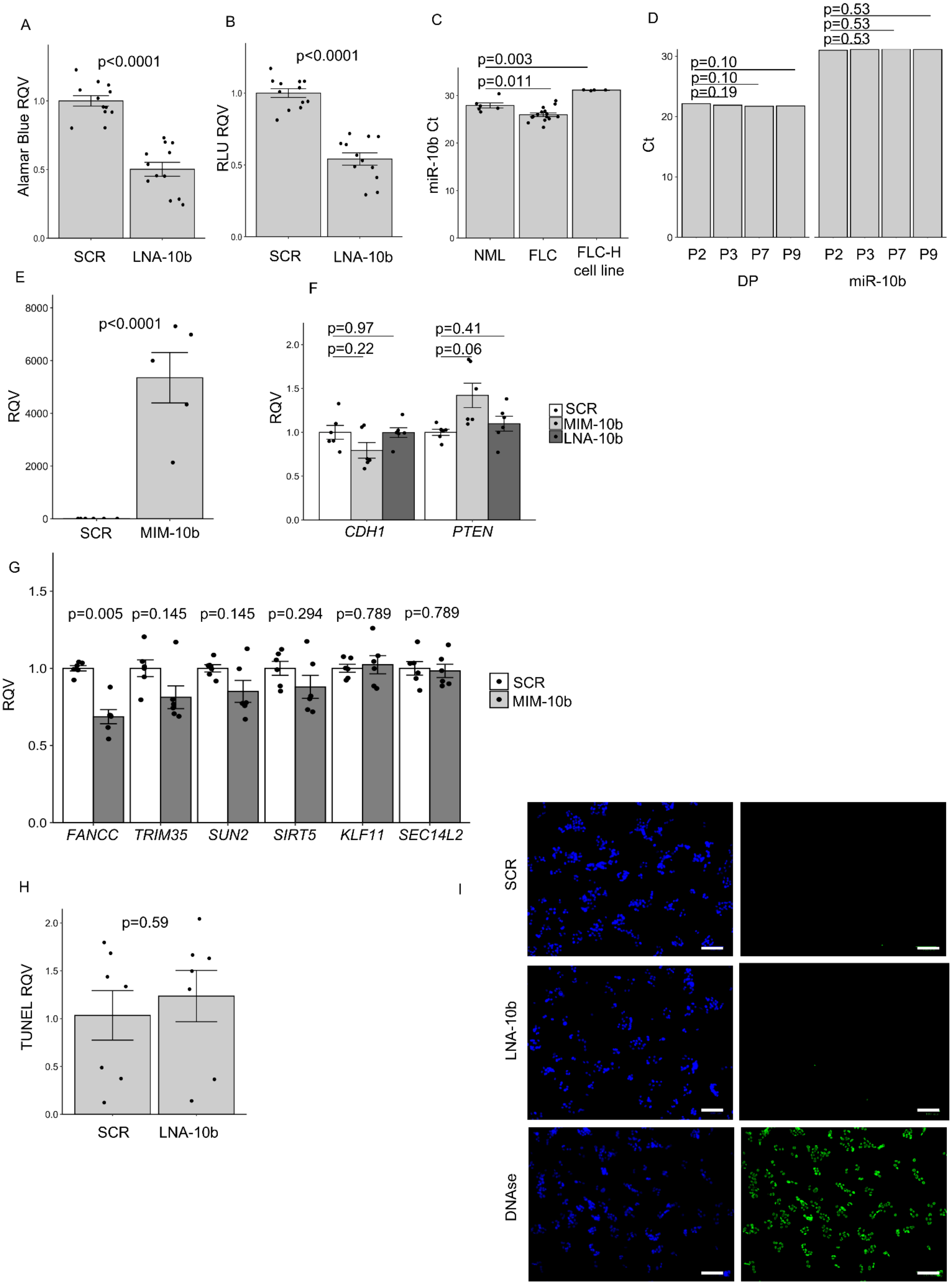

Supplemental Figure 5. (A) Alamar blue absorbance and **(B)** luciferase relative light unit (RLU) assays in FLC-C cells after treatment with miR-10b LNA is shown as RQV compared to the negative control (scrambled sequence) 2 days after 100nM treatment. **(C)** Quantitative PCR cycle threshold (Ct) values for miR-10b expression in non-malignant liver (NML), fibrolamellar carcinoma (FLC), and the FLC-H cell line. **(D)** Quantitative PCR cycle threshold (Ct) values for DP and miR-10b expression in FLC-H cell passage 2, 3, 7, and 9 (P2-P9). **(E)** Quantitative PCR showing the RQV of miR-10b in FLC-H cells 6 days after 500nM treatment with miR-10b mimic (MIM-10b) or scrambled sequence compared to mock (2 trials, n=6 each condition). **(F)** Quantitative PCR showing the RQV of *CDH1* and *PTEN* in FLC-H cells 6 days after 500nM treatment with miR-10b mimic (MIM-10b), miR-10b inhibitor (LNA-10b), or scrambled sequence compared to mock (2 trials, n=6 each condition). **(G)** Quantitative PCR showing the RQV of *FANCC, TRIM35, SUN2, SIRT5, KLF11,* and *SEC14L2* in FLC-H cells after 6 days of 500nM miR-10b MIM treatment compared to the negative control (2 trials, n=6 in each condition). **(H)** TUNEL assay in FLC-H cells 6 days after 500nM treatment with miR-10b LNA compared to the negative control shown as RQV (2 trials, n=7 in each condition). **(I)** Representative DAPI and TUNEL stained images show total and apoptotic cells in scramble and miR-10b LNA treated cells. After fixation a subset of cells were treated with DNase to provide a positive control for the staining reaction. Scale bars equal 100 µM. In all assays, each dot represents the average signal across technical replicates for a single biological replicate. *P*-values calculated by two-tailed Student's t-test. *P*-values reported in panels C, D, F and G were adjusted for multiple testing correction post-hoc by benjamini-hochberg method.
