# Supplementary material for: Multiomic analysis of microRNA-mediated regulation reveals a proliferative axis involving miR-10b in fibrolamellar carcinoma
Source: JCI Insight. 2022 Jun 8;7(11):e154743. doi: 10.1172/jci.insight.154743 (PMC9220943; doi:10.1172/jci.insight.154743)
Supplement: Supplemental table 2 [file jciinsight-7-154743-s132.pdf]

```
Sample,RIN,Included in study,Total Reads,Trimmed Reads,Percent Trimmed
Reads,Too Short Reads,Percent Too Short,Exact Match Reads,Percent
Exact Matches,Mismatch Reads,Percent Mismatched,Mapped Reads,Percent
Mapped,miR Mapped Reads,Percent miR Mapped,tRNA Mapped Reads,Percent
tRNA Mapped,yRNA Mapped Reads,Percent yRNA Mapped
FLC01_LNBS,NA,Yes,38565825,21368854,55.41,6104604,15.83,15196485,71.12
,6172369,28.88,19042689,89.11,8051694,42.28,2751932,14.45,89263,0.47
FLC04_CDHM,NA,Yes,52252452,43533173,83.31,5522204,10.57,33131501,76.11
,10401672,23.89,38005210,87.3,5297001,13.94,23319355,61.36,120285,0.32
FLC06_DZIS,8.3,Yes,37916979,30321242,79.97,3126857,8.25,23460005,77.37
,6861237,22.63,26050512,85.92,5312246,20.39,16261406,62.42,65212,0.25
FLC06_GMKX,NA,Yes,43551830,30613909,70.29,5982522,13.74,23583706,77.04
,7030203,22.96,26963741,88.08,7558949,28.03,12922476,47.93,67887,0.25
FLC06_SLVP,7.3,Yes,39201032,31957001,81.52,5738505,14.64,24430425,76.4
5,7526576,23.55,27969546,87.52,8943033,31.97,13284063,47.49,79635,0.28
FLC06_UFNW,7.2,No,31709763,26157354,82.49,3313014,10.45,20272917,77.5,
5884437,22.5,22446171,85.81,3503175,15.61,14497534,64.59,55461,0.25
FLC07_LFOC,NA,Yes,43217504,34130339,78.97,5103124,11.81,27582868,80.82
,6547471,19.18,30627251,89.74,4122974,13.46,21807288,71.2,45081,0.15
FLC09_MUOD,7.5,Yes,51896813,26939803,51.91,21408169,41.25,19430821,72.
13,7508982,27.87,23255938,86.33,6673945,28.7,6937117,29.83,68082,0.29
FLC09_TZOG,6.2,Yes,43494156,21044427,48.38,19652178,45.18,14050750,66.
77,6993677,33.23,18495395,87.89,9198707,49.74,1330523,7.19,42708,0.23
FLC12_CTTF,5.2,Yes,43042357,24234004,56.3,17804563,41.37,17615612,72.6
9,6618392,27.31,20874089,86.14,3980946,19.07,6005324,28.77,44581,0.21
FLC13_QQEI,5.8,Yes,46180849,32107007,69.52,9394356,20.34,22719351,70.7
6,9387656,29.24,27872194,86.81,7395884,26.53,4509898,16.18,108496,0.39
FLC15_AWTJ,6.7,Yes,41846226,29891335,71.43,4677516,11.18,22116200,73.9
9,7775135,26.01,25243251,84.45,4860837,19.26,14918846,59.1,122071,0.48
FLC17_WCKN,4.9,Yes,38087717,22147253,58.15,3601227,9.46,16239652,73.33
,5907601,26.67,19708379,88.99,8669839,43.99,5719646,29.02,141928,0.72
FLC18_FXYQ,9.6,Yes,38782638,18250022,47.06,6814804,17.57,12665413,69.4
,5584609,30.6,15432715,84.56,3692315,23.93,6066072,39.31,111643,0.72
FLC18_MKZC,8.5,Yes,42785259,24387944,57,6721829,15.71,16730062,68.6,76
57882,31.4,19302822,79.15,4362098,22.6,9183423,47.58,72884,0.38
FLC18_QFZD,8.7,No,37268074,31010874,83.21,2943956,7.9,22829224,73.62,8
181650,26.38,26154900,84.34,2433699,9.3,17799303,68.05,45693,0.17
FLC18_WSVW,8.5,No,35460757,28506931,80.39,3098656,8.74,20689469,72.58,
7817462,27.42,23779750,83.42,3573964,15.03,14911442,62.71,63021,0.27
FLC20_ZDNV,9.3,Yes,21408102,14365365,67.1,4866022,22.73,11683533,81.33
,2681832,18.67,13184780,91.78,6464853,49.03,2657343,20.15,228585,1.73
FLC23_RVBN,8.3,No,39329914,13283745,33.78,5506909,14,8945265,67.34,433
8480,32.66,11488320,86.48,5368114,46.73,762213,6.63,100318,0.87
FLC24_DJZW,4.7,Yes,36480322,31527562,86.42,3343929,9.17,28674857,90.95
,2852705,9.05,29784369,94.47,2422694,8.13,21346321,71.67,158174,0.53
FLC25_BCRB,6.9,No,33599054,19314385,57.48,3899628,11.61,15061545,77.98
,4252840,22.02,17494095,90.58,9286090,53.08,4341840,24.82,52524,0.3
FLC25_UYHR,6.8,Yes,38344117,21587758,56.3,3492885,9.11,16497236,76.42,
5090522,23.58,19661287,91.08,10053009,51.13,5114074,26.01,88892,0.45
FLC26_ICBQ,4.8,Yes,37004899,23704827,64.06,4520400,12.22,17162189,72.4
```

,6542638,27.6,21410238,90.32,11466779,53.56,4645978,21.7,86221,0.4
FLC26_OAOE,8.6,Yes,35401030,22488764,63.53,8772998,24.78,16704319,74.2
8,5784445,25.72,20182607,89.75,11160210,55.3,2936842,14.55,98142,0.49
FLC26_YJEE,9,Yes,39917718,20076432,50.29,5611201,14.06,14728049,73.36,
5348383,26.64,17815925,88.74,9691886,54.4,2708360,15.2,98119,0.55
FLC27_BDCH,8.1,Yes,62872615,45731105,72.74,11612799,18.47,34607034,75.
68,11124071,24.32,40238902,87.99,8428971,20.95,21684023,53.89,129779,0
.32
FLC27_BWSX,6.3,Yes,56743245,41829397,73.72,10039437,17.69,30845885,73.
74,10983512,26.26,37057552,88.59,15593516,42.08,12248329,33.05,128545,
0.35
FLC27_XDGP,6.4,Yes,69498231,52764180,75.92,14640623,21.07,39655775,75.
16,13108405,24.84,47165948,89.39,12784948,27.11,23099862,48.98,146835,
0.31
FLC28_RKXK,7.8,Yes,32514031,20124901,61.9,4446290,13.67,14204565,70.58
,5920336,29.42,17312083,86.02,8118066,46.89,3727905,21.53,84719,0.49
FLC29_QLXW,8.9,Yes,30469829,21399081,70.23,2643726,8.68,16375236,76.52
,5023845,23.48,18930986,88.47,8146463,43.03,7264427,38.37,45678,0.24
FLC30_KPKS,7.4,Yes,32671190,25039169,76.64,4535228,13.88,19075183,76.1
8,5963986,23.82,22693797,90.63,7582100,33.41,10227601,45.07,53935,0.24
FLC31_OTOK,7.8,Yes,32215798,23430981,72.73,7812420,24.25,19536601,83.3
8,3894380,16.62,21396338,91.32,8091242,37.82,4329229,20.23,413524,1.93
FLC32_UOTX,7.7,Yes,36188677,30169312,83.37,4808271,13.29,26355007,87.3
6,3814305,12.64,28560417,94.67,16716192,58.53,6938032,24.29,69929,0.24
FLC33_NYTS,6.7,Yes,36263797,24693502,68.09,5751989,15.86,19693919,79.7
5,4999583,20.25,22244171,90.08,12559177,56.46,4013481,18.04,84155,0.38
FLC34_PMVV,6.2,Yes,40306757,34559421,85.74,5170534,12.83,29967941,86.7
1,4591480,13.29,33248085,96.21,17548082,52.78,10567352,31.78,96316,0.2
9
FLC34_YROP,7.2,Yes,36001842,30612806,85.03,2995097,8.32,23843170,77.89
,6769636,22.11,26507860,86.59,6241403,23.55,15898369,59.98,145979,0.55
FLC55_T,7.6,Yes,49413354,31892883,64.54,4417432,8.94,25877053,81.14,60
15830,18.86,28449794,89.2,3231017,11.36,19807346,69.62,114307,0.4
FLC56_N,7.4,Yes,51638161,39736531,76.95,3962370,7.67,32248346,81.16,74
88185,18.84,36925539,92.93,14339464,38.83,15587590,42.21,118341,0.32
FLC56_T,7.4,Yes,65497143,48292691,73.73,7859770,12,37058895,76.74,1123
3796,23.26,42057296,87.09,4583370,10.9,26156933,62.19,113697,0.27
FLC57_T,5.7,Yes,49895710,38570222,77.3,4009559,8.04,29663214,76.91,890
7008,23.09,34300128,88.93,13307670,38.8,15264434,44.5,154596,0.45
FLC58L_T,8.2,Yes,63584856,41099805,64.64,9400222,14.78,32885707,80.01,
8214098,19.99,36794449,89.52,14761691,40.12,12599694,34.24,291617,0.79
FLC82_T,7.7,Yes,31594228,29234248,92.53,1948930,6.17,27095821,92.69,21
38427,7.31,28377073,97.07,7117447,25.08,17852006,62.91,51054,0.18
FLC83_N,4,Yes,32177677,28398157,88.25,3522774,10.95,25649164,90.32,274
8993,9.68,27485264,96.79,5402347,19.66,17626137,64.13,50257,0.18
FLC83_T,6.3,Yes,59761624,44814883,74.99,5202712,8.71,36731100,81.96,80
83783,18.04,41087292,91.68,7827022,19.05,23970153,58.34,198392,0.48
FLC84_T,4.2,Yes,65904866,53540226,81.24,4386425,6.66,43428408,81.11,10
111818,18.89,50018643,93.42,6426845,12.85,32441134,64.86,176843,0.35
FLC85_T,8.6,Yes,58079488,44412223,76.47,6179742,10.64,36731542,82.71,7

680681,17.29,40196656,90.51,8790982,21.87,23714633,59,149427,0.37
FLC87_N,6.3,Yes,55850541,33062669,59.2,4955513,8.87,24702512,74.71,836
0157,25.29,29455656,89.09,7464758,25.34,13510536,45.87,474675,1.61
FLC88_T,6.6,Yes,59134504,47087511,79.63,3048729,5.16,38019128,80.74,90
68383,19.26,43659678,92.72,6440751,14.75,28612787,65.54,159446,0.37
FLC89_N,8.1,Yes,50320961,39623428,78.74,2423697,4.82,33788047,85.27,58
35381,14.73,37871268,95.58,8004867,21.14,22650528,59.81,201779,0.53
FLC89_T,6.2,Yes,46138663,27741008,60.13,6800459,14.74,22389221,80.71,5
351787,19.29,23696259,85.42,2277136,9.61,13879057,58.57,395461,1.67
FLC90_N,6.3,Yes,55024227,46061844,83.71,3305553,6.01,40086729,87.03,59
75115,12.97,43957158,95.43,8337575,18.97,27582678,62.75,598458,1.36
FLC90_T,4.3,Yes,39210305,35456575,90.43,2026286,5.17,29251582,82.5,620
4993,17.5,33874128,95.54,1634903,4.83,23143164,68.32,489968,1.45
